# A *Porphyromonas gingivalis* hypothetical protein controlled by the type I-C CRISPR-Cas system is a novel adhesin important in virulence

**DOI:** 10.1128/msystems.01231-23

**Published:** 2024-02-07

**Authors:** Muhammad Irfan, Jose Solbiati, Ana Duran-Pinedo, Fernanda Godoy Rocha, Frank C. Gibson, Jorge Frias-Lopez

**Affiliations:** 1 Department of Oral Biology, College of Dentistry, University of Florida, Gainesville, Florida, USA; 2 Department of Periodontology, College of Dentistry, University of Florida, Gainesville, Florida, USA; California State University, Stanislaus, Turlock, California, USA

**Keywords:** *Porphyromonas gingivalis*, hypothetical protein, adhesin, *Galleria mellonella*, CRISPR, TLR2/4, DUF2807

## Abstract

The ability of many human pathogens to infect requires their ability to adhere to the host surfaces as a first step in the process. *Porphyromonas gingivalis,* a keystone oral pathogen*,* uses adhesins to adhere to the surface of the gingival epithelium and other members of the oral microbiome. In a previous study, we identified several proteins potentially linked to virulence whose mRNA levels are regulated by CRISPR-Cas type I-C. Among those, PGN_1547 was highly upregulated in the CRISPR-Cas 3 mutant. PGN_1547 is annotated as a hypothetical protein. Employing homology searching, our data support that PGN_1547 resembles an auto-transporter adhesin of *P. gingivalis* based on containing the DUF2807 domain. To begin to characterize the function of PGN_1547, we found that a deletion mutant displayed a significant decrease in virulence using a *Galleria mellonela* model. Furthermore, this mutant was significantly impaired in forming biofilms and attaching to the macrophage-like cell THP-1. Luminex revealed that the PGN_1547 mutant elicited a less robust cytokine and chemokine response from THP-1 cells, and TLR2 predominantly sensed that recombinant PGN_1547. Taken together, these findings broaden our understanding of the toolbox of virulence factors possessed by *P. gingivalis*. Importantly, PGN_1547, a hypothetical protein, has homologs in another member of the order Bacteroidales whose function is unknown, and our results could shed light on the role of this family of proteins as auto-transport adhesins in this phylogenetic group.

**IMPORTANCE:**

Periodontal diseases are among humans’ most common infections, and besides their effect on the oral cavity, they have been associated with systemic inflammatory conditions. Among members of the oral microbiome implicated in the development of periodontitis, *Porphyromonas gingivalis* is considered a keystone pathogen. We have identified a new adhesin that acts as a virulence factor, PGN_1547, which contains the DUF2807 domain, which belongs to the putative auto-transporter adhesin, head GIN domain family. Deletion of this gene lowers the virulence of *P*. *gingivalis* and impacts the ability of *P*. *gingivalis* to form biofilm and attach to host cells. Furthermore, the broad distribution of these receptors in the order Bacteroidales suggests their importance in colonization by this important group of organisms.

## INTRODUCTION

*Porphyromonas gingivalis* is a Gram-negative anaerobic oral pathogen associated with chronic periodontitis and atherosclerosis (1–4). *P. gingivalis* is a significant pathogen thought to drive dysbiotic biofilm formation that causes periodontitis, a polymicrobial disease resulting from the coordinated action of a complex microbial community and dysregulates host immune responses, leading to chronic inflammation and the destruction of soft and hard tissues supporting the teeth. Polymicrobial synergistic interactions among community inhabitants raise the pathogenic community potential, and *P. gingivalis*, acting as a keystone pathogen, can drive the transition of a commensal community to a pathogenic one, even at low abundance and is thought to stabilize the dysbiotic microbiota associated with the disease state (5–7). Thus, controlling *P. gingivalis* is essential to maintaining a healthy subgingival biofilm. However, the fundamental mechanisms underlying the transition to periodontitis still need to be fully understood.

The first step in colonizing the oral cavity is the adhesion of the microorganisms to the teeth or mucosal surfaces (8, 9). The adhesion of bacteria to the varied oral surfaces and other microbial community members is usually mediated by adhesins on the surface of bacteria and by receptors on the oral surface or adhesins from other organisms (10–12). Microbial adhesins are found as cell wall components or are associated with cell structures, such as capsules or fimbriae (13).

*P. gingivalis* pili are filamentous structures located on the *P. gingivalis* surface, which enhance bacterial adhesion to multiple types of surfaces, such as the extracellular matrix, host cells, and other bacteria, and form biofilm (14). *P. gingivalis* possess at least two types of fimbriae, the major FimA and the minor Mfa; both regulate bacterial dependence on various molecules and oral substrates and are essential for biofilm formation (15, 16). Adhesins not only play a role in colonization but are also crucial in the modulation of the host’s immune response. *P. gingivalis* long fimbrial proteins are capable of activating human gingival epithelial cells through Toll-like receptor (TLR)-2 and significantly upregulating IL-8 expression and NF-κB activation, which are involved in bone resorption (9, 17). The minor fimbriae, Mfa, enhances the bone resorption of osteoclasts by producing IL-1β, TNF-α, and IL-6 and promotes the differentiation of osteoclast precursor cells (14).

Previously, our lab demonstrated that CRISPR-Cas systems control virulence in *P. gingivalis*. We showed the importance of type I-C system of *P. gingivalis* ATCC 33277 by deleting a nuclease essential in this kind of system, Cas3 (18). In this study, we assessed the effect of the deletion of the nuclease in the transcriptome of *P. gingivalis* to identify potential genes controlled by such systems. Among the genes most highly upregulated in the *cas*3 mutant was the hypothetical protein PGN_1547. This critically over-expressed gene in Δ*cas3* mutant had been previously identified as essential in fitness in two model systems used to study virulence in *P. gingivalis* ATCC 33277 (19). PGN_1547 is annotated as a hypothetical protein containing a DUF2807 domain belonging to the trimeric auto-transporter adhesins (TAAs) superfamily. TAAs are essential virulence factors in Gram-negative pathogens (20, 21).

The present study demonstrates that this hypothetical protein acts as an essential virulence factor, as shown in a *Galleria mellonella* model for virulence. Moreover, we demonstrated that PGN_1547 is an adhesin essential in adhesion to THP-1 cells. The purified recombinant *P. gingivalis* PGN_1547 protein showed induction of TLR signaling pathways primarily through TLR-2, thus modulating the immune response. The implication that PGN_1547 may be an essential virulence factor in *P. gingivalis* is discussed.

## RESULTS

### Deletion of the hypothetical protein PGN_1547 decreases virulence in *P. gingivalis* ATCC 33277

**Fig 1:**
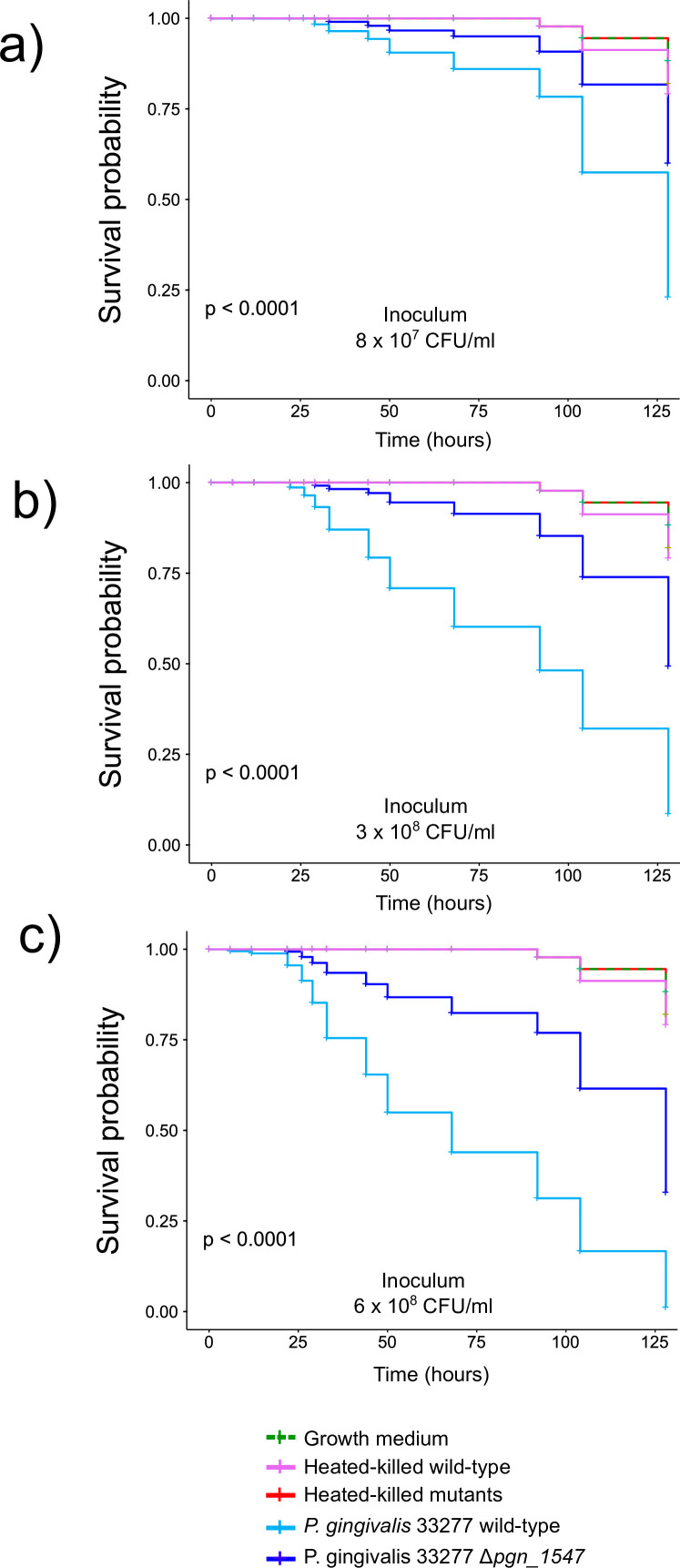
Survival curves in *Galleria mellonella*. Kaplan-Meier survival curves were determined. *G. mellonella* larvae were injected with the *P. gingivalis* ATCC 33277 wild type and the *Δpgn_1547* mutant. Three different dilutions of the inocula were tested. Inoculum per worm in the wild type and *Δpgn_1547* were (a) 8 × 10^7^ CFU/mL, (b) 3 × 10^8^ CFU/mL, and (c) 6 × 10^8^ CFU/mL. Survival was monitored for 125 hours in wild type and *Δpgn_1547* mutant. Larvae were also inoculated with three negative controls: tryptic soy broth (TSB) medium where bacteria grew, TSB medium plus the wild-type strain (heat killed), and TSB medium plus the mutant strain (heat killed).

Our previous study found that protein PGN_1547 was highly upregulated in the Δ*cas*3 mutant from the same type I-C CRISPR-Cas system in *P. gingivalis* (18). The deletion of *cas*3 increased virulence. Thus, we were expecting a decrease in virulence when we deleted PGN_1547. To directly assess the contribution of Δ*pgn_1547* in the virulence of *P. gingivalis*, we challenged groups of *G. mellonella* larvae by injection with different dilutions of *P. gingivalis* wild-type and Δ*pgn_1547* knockout strains. We found significantly lower mortality (*P* < 0.0001) for the groups of worms infected with the Δ*pgn_1547* mutant, with 50% mortality within the first 120 hours when larvae were injected with 8 × 10^7^ CFU (colony-forming unit)/mL (Fig. 1a) and less than 50% mortality when larvae were injected with 3 × 10^8^ (Fig. 1b) and 6 × 10^8^ CFU/mL (Fig. 1c), respectively. In contrast, 60% of the larvae infected with the *P. gingivalis* wild-type strain died within the first 60 hours when larvae were injected with 6 × 10^8^ CFU/mL (Fig. 1c). No larva mortality was observed in the control groups until 5 days after inoculation, and larvae injected with heat-killed mutants and with growth medium followed the same survival profiles (Fig. 1a through c).

### Deleting the hypothetical protein, PGN_1547, in *P. gingivalis* ATCC 33277 decreases the ability to produce biofilms and attach to THP-1 cells

**Fig 2:**
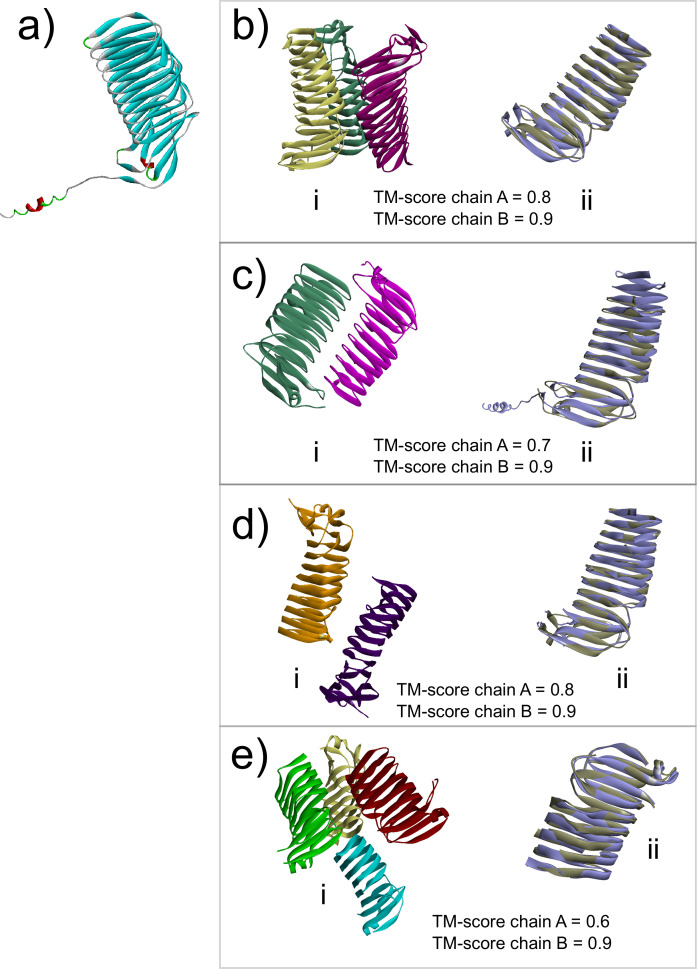
3D protein models and alignments. Models were downloaded from the RCSB Protein Data Bank (PDB) (https://www.rcsb.org/). (a) 3D model of PGN_1547 as predicted by alphafold2 (22, 23) at the AlphaFold Protein Structure Database (https://alphafold.ebi.ac.uk/). (b) (i) PDB model for the putative adhesin (YP_001304413.1) from *Parabacteroides distasonis* ATCC 8503 (3LJY.pdb) and (ii) TM-align alignment with PGN_1547. (c) (i) PDB model for the putative adhesin (BF0245) from *Bacteroides fragilis* NCTC 9343 (3PET.pdb) and (ii) TM-align alignment with PGN_1547. (d) (i) PDB model for putative adhesin (PARMER_02777) from *Parabacteroides merdae* ATCC 43184 (4OPW.pdb) and (ii) TM-align alignment with PGN_1547. (e) (i) PDB model for putative adhesin (BACEGG_01763) from *Bacteroides eggerthii* DSM 20697 (4YGU.pdb) and (ii) TM-align alignment with PGN_1547.

PGN_1547 is a gene with an unknown function. Based on homology searches, we identified that PGN_1547 is a DUF2807-containing domain protein with high similarity to other known auto-transporter adhesins (20, 21). The 3D structure of PGN_1547 was predicted by alphafold2 (22, 23) (Fig. 2a), and we compared it to known resolved adhesin structures that contain the DUF2807 domain. Four structures have been resolved; the putative adhesin (YP_001304413.1) from *Parabacteroides distasonis* ATCC 8503 (3LJY.pdb), the putative adhesin (BF0245) from *Bacteroides fragilis* NCTC 9343 (3PET.pdb), the putative adhesin (PARMER_02777) from *Parabacteroides merdae* ATCC 43184 (4OPW.pdb), and a putative adhesin (BACEGG_01763) from *Bacteroides eggerthii* DSM 20697 (4YGU.pdb). All of these putative adhesins contain more than one subunit. We aligned PGN_1547 to all four using the TM-align server (24) and found high structural homology (Fig. 2). A TM-score >0.5 indicates an identical folding pattern (24). In all comparisons, we observed higher values than 0.5.

**Fig 3:**
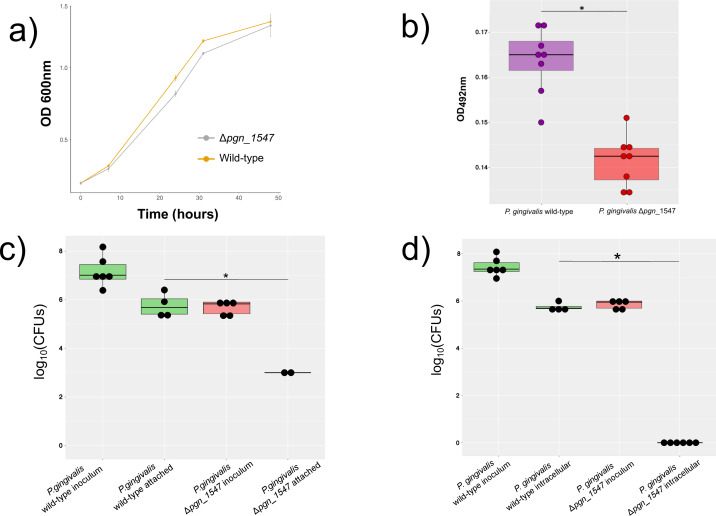
Effect of deletion of PGN_1547 in attachment properties of *P. gingivalis* ATCC 33722. (**a**) Growth curves of *P. gingivalis* ATCC 33277 and Δ*pgn_1547* mutant growing in 20% heat-inactivated human serum supplemented with 1 µg/mL menadione and 5 µg/mL hemin. (**b**) Biofilm growth on microtiter plates measured by safranin staining. (**c**) Number of CFUs recovered from THP-1 attachment. (**d**) Percentage of adhered bacteria to THP-1 cells. * *P*-value < 0.05.

Adhesins play an essential role in the attachment of *P. gingivalis* to host cells and other organisms that are members of the oral biofilm (8), thus being an essential part of the pathogen arsenal of virulence factors. Therefore, wild-type and mutant biofilm screenings were carried out in polystyrene plates to assess the effect of the deletion on biofilm formation. The deletion of PGN_1547 had no significant impact on growth when the *P. gingivalis* strains were grown planktonically, with similar doubling times of 9 hours in the case of the wild type and 9.6 hours in the case of the mutant (Fig. 3a; Fig. S1). Moreover, there were no significant differences in the slope values of the linear growth phase (*P* = 0.7271 by analysis of variance, ANOVA). However, we found that the ability to form biofilm was significantly decreased in the Δ*pgn_1547* mutant when compared to the wild type (Fig. 3b). Another critical aspect of adhesins in the pathogenesis of *P. gingivalis* is its ability to be used to attach to host cells (8). Therefore, we assessed the effect of the mutation on the ability to attach to and invade THP-1 cells and unexpectedly observed that viable Δ*pgn_1547* mutant bacteria did not readily adhere to THP-1 cells (Fig. 3c), and even fewer of the mutant bacteria were found inside THP-1 cells after 6 hours of infection (Fig. 3d).

### Host-pathogen transcriptomes show a decrease in the induction of immune response in *P. gingivalis* Δ*pgn_1547*

Next, we wanted to characterize the early phase transcriptional differences in host-pathogen interactions using THP-1 cells as a model of immune phagocytes. Therefore, we assessed the effect of this Δ*pgn_1547* deletion in host response and the bacterium attaching and invading TPH-1 cells at 2 and 6 hours of *P. gingivalis* cell infection. At 2 hours, we found 115 host genes differentially expressed and 557 *P*. *gingivalis* genes using PATRIC annotation (25) (Tables S2 and S3).

**Fig 4:**
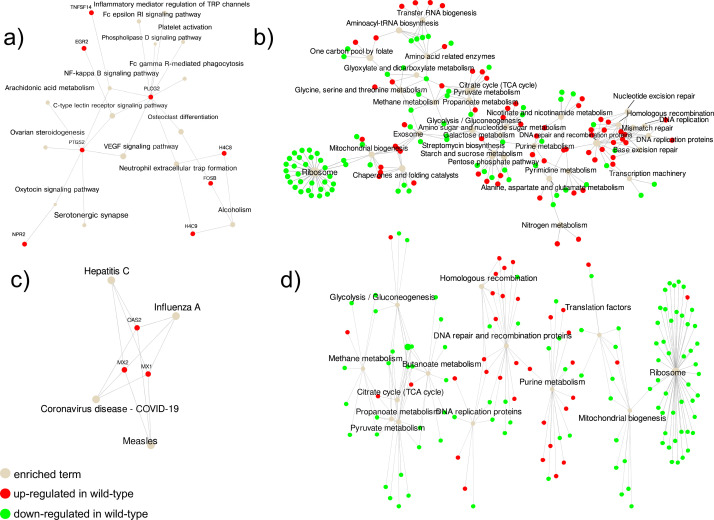
pathfindR enrichment of KEGG pathways in THP-1 and *P. gingivalis* during cell infection. Bubble chart of enrichment results grouped by clusters. The bubble size indicates the number of differentially expressed genes in the given pathway. In red and green are up and downregulated I genes in the wild-type infection experiment. (**a**) Enrichment of KEGG terms in THP-1 cells at 2 hours of incubation. (**b**) Enrichment of KEGG terms in *P. gingivalis* at 2 hours of incubation. (**c**) Enrichment in THP-1 cells at 6 hours of incubation. (**d**) Enrichment in *P. gingivalis* at 6 hours of incubation.

We first compared whole transcriptome profiles in THP-1 cells infected with the wild type and the Δ*pgn_1547* mutant. The significant differences in gene expression profiling observed were due to the incubation time rather than the strain co-cultured with the cells. At 2 hours, profiles of expression of THP-1 infected with the two strains were more similar than their corresponding profiles at 6 hours (Fig. S2a). At 6 hours, the number of differentially expressed genes was lower in the host (40 differentially expressed genes) but higher in *P. gingivalis* (1,008 differentially expressed genes, using PATRIC annotation [25]) (Tables S2 and S3). All genes in the host’s enriched pathways at 2 and 6 hours showed upregulated genes in THP-1 cells infected by the wild type (Fig. 4a and c). Enriched pathways at 2 and 6 hours were associated with different metabolic activities. We found that at 2 hours of infection, several immune response pathways were associated with infection by the mutant, such as neutrophil extracellular trap formation, NF-kappa B signaling, and Fc gamma R-mediated phagocytosis were upregulated in the wild type compared to the mutant (Fig. 4a). Additionally, osteoclast differentiation was also induced at 2 hours of infection. By contrast, genes that were upregulated at 6 hours were induced by interferons and antagonized the replication process of several different RNA and DNA viruses (MX1 and MX2) (Fig. 4c).

In the case of *P. gingivalis*, differences in whole transcriptome profiles between the wild type and the PGN_1547 mutant were observed at 2 hours of incubation with THP-1 cells (Fig. S2b). While the wild type did not show a significant shift in transcriptome profiles between 2 and 6 hours of incubation, Δ*pgn_1547* mutant showed a significant shift between the two time points (Fig. S2b). When looking at the physiological activities impacted, the wild type was less active at 2 and 6 hours than the mutant, as indicated by the downregulated expression of genes associated with ribosome synthesis (Fig. 4b and d). Differences in gene expression were more marked at 2 hours, probably due to an entrance in a less active state in both the mutant and the wild type at 6 hours. Genes associated with DNA repair were upregulated in the wild type compared to the mutant (Fig. 4b and d). Although many genes specific to inflammatory pathways were differentially expressed between wild type and the Δ*pgn_1547* mutant, we unexpectedly did not find significant statistical differences but rather trends in the expression of those genes that encode the cytokines and chemokines that were represented in the multiplex immunoassays (Fig. S3).

### Cytokine and chemokine response from THP-1 to *P. gingivalis* is reduced in Δ*pgn_1547* mutant

**Fig 5:**
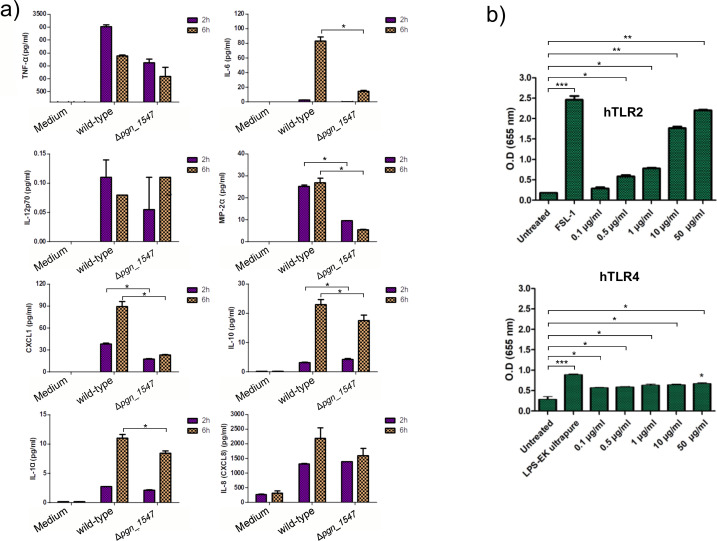
Influence on soluble immune mediators and activation of TLR2/TLR4. (**a**) After 2 and 6 hours of incubation with the *P. gingivalis* wild-type strain and Δ*pgn_1547* mutant, at a multiplicity of infection of 100, supernatant fluids from THP-1 cell culture were analyzed. Levels of different cytokines were measured by multiplex immunoassay. Medium only (unchallenged control). Data are presented as means ± standard errors of the means (*n* = 3 independent experiments). (**b**) The purified PGN_1547 protein of *P. gingivalis* induces hTLR2 and hTLR4 activation. HEK-Blue hTLR2 cells were incubated with purified PGN_1547 protein at different concentrations (0.1, 0.5, 1, 10, and 50 µg/mL), FSL-1 (positive control), or left untreated (negative control). Levels of hTLR2 activation were determined by measuring the absorbance at 655 nm. HEK-Blue hTLR4 cells were incubated with purified PGN_1547 protein at different concentrations (0.1, 0.5, 1, 10, and 50 µg/mL), LPS-EK Ultrapure (positive control), or left untreated (negative control). Levels of hTLR4 activation were determined by measuring the absorbance at 655 nm. Experiments were performed in triplicates, and data are shown as mean ± s.d. The average of three independently performed experiments is shown. One-way ANOVA determined statistical significance.

As inflammation is a crucial driver of the bacterium-elicited tissue destruction that characterizes the periodontal disease, we assessed the effect of Δ*pgn_1547* mutant on the cytokine profiles of THP-1 macrophage-like cells. Using Luminex multiplex immunoassay, we measured cell-culture supernatant fluid levels of the cytokines at 2 and 6 hours following infection. Overall, we observed a trend of decrease in cytokine and chemokine production levels in the THP-1 cells infected with the mutant as compared with wild type (Fig. 5a). After 2 hours of co-culture, we observed that only CXCL1, MIP-2-a, and IL-10 were significantly reduced in expression from THP-1 cells cultured with Δ*pgn_1547* mutant compared with wild-type *P. gingivalis*. At 6 hours, we found that cell-culture supernatant fluid levels of IL-6, IL-1α, IL-10, CXCL1, and MIP-2-α were significantly lower in the mutant (Fig. 5a).

### Toll-like receptor 2 and 4 activation is PGN_1547 dependent

Finally, we investigated the mechanism of innate immune sensing of PGN_1547 by the host. We focused on TLR-2 and TLR4 due to their essential role in sensing and subsequent cytokine and chemokine production in response to *P. gingivalis* challenge (26–29). First, HEK 293 TLR2 and HEK TLR4 reporter cells were infected with wild-type *P. gingivalis* 33277 and Δ*pgn_1547* mutant as part of this assay. Measurement of secreted embryonic alkaline phosphatase (SEAP) revealed that activation of investigated receptors gave a colorimetric reaction that developed over time and was measured at 655 nm absorbance. The preceding experiments demonstrate that the activating effect of purified PGN_1547 is TLR-dependent, likely via MyD88 (both TLR2 and TLR4) or possibly some signaling via TRIF (TLR4 only). Therefore, PGN_1547 was tested at 0.1, 0.5, 1, 10, and 50 µg/mL concentrations of recombinant PGN_1547, and we found that PGN_1547 predominantly activated HEK-Blue hTLR2 although some very minimal HEK-Blue hTLR4 sensing may be present (Fig. 5b).

## DISCUSSION

Adhesins are essential in colonizing oral surfaces and are important virulence factors in oral pathogens (30, 31). In *P. gingivalis* fimbriae (long and short) and gingipain, hemagglutinin domains are considered the most critical adhesins involved in the colonization of the subgingival epithelium and co-aggregation with other organisms (30, 32, 33). Fimbriae are thin, filamentous structures synthesized by most strains of *P. gingivalis*, promoting biofilm formation, bacterial adhesion to host cells, and bacterial invasion into cells (34). *P. gingivalis* has two types of fimbriae: long and short. Long fimbriae (FimA is its primary component) are involved in the invasion of host cells, triggering the host’s immune response by binding to TLR2 (17, 35), while short fimbriae (whose major protein subunit is Mfa1) bind to *Streptococcus gordonii* (30, 36) and helps differentiate osteoclast precursor cells into osteoclasts and enhance bone resorption and cellular adhesion (14, 27, 37). In the present study, we identified the hypothetical protein PGN_1547 as a new adhesin and examined its functional role in the virulence of *P. gingivalis* ATCC 33277.

The most direct evidence of the effect on virulence in the mutant strain came from our experiments using *G. mellonella* as an infection model. Previous work with several microbial pathogens demonstrated a positive correlation between this model’s results and other mammalian disease models (38–40). The innate immune responses of *G. mellonella* are comparable with vertebrate innate immune responses and involve recognizing the bacteria and producing antimicrobial molecules (41). Moreover, the *G. mellonella* model is suitable for studying pathogenesis and immune responses in human oral pathogens (42), including *P. gingivalis* (43, 44). Our data showed that an in-frame deletion mutant of the *pgn_1547* gene significantly decreases the virulence of the mutated strain.

Our analysis reveals that the PGN_1547 protein is structurally similar to other DUF2807 domain-containing protein parts of the putative auto-transporter adhesin, head GIN domain family members. According to the CDD/SPARCLE classification of proteins, this family probably belongs to the superfamily of auto-transporter adhesins and, as such, would place these structures as essential virulence factors in Gram-negative pathogens (45). Supporting the hypothesis that this protein acts as a TAA adhesin in *P. gingivalis*, we found that its putative 3D structure is identical to other known structures of this type of adhesin. These adhesins were found to belong to members of the order Bacteroidales. Two of the structures are dimers, while the others are one trimer and a tetramer.

Interestingly, downstream of PGN_1547, we find another hypothetical protein (PGN_1548) with an identical 3D structure as PGN_1547. However, PGN_1547 and PGN_1548 are not in an operon, and each protein is controlled by its own promoter. Thus, one hypothesizes that those two proteins are part of the final outer membrane structure acting as adhesins.

We obtained direct evidence of the role of this protein in adhesion from two different sources. First, as could be expected of an adhesin, biofilm formation was statistically decreased in the Δ*pgn_1547* strain. Biofilm formation is one of the essential virulence determinants of *P. gingivalis*. It is associated with resistance to the response of the host immune system (34, 35) and co-aggregation with other members of the oral microbiome (30). Second, we found that the mutant was significantly impaired in its ability to attach to and persist in THP-1 macrophage-like cells. This finding is important as *P. gingivalis* can invade epithelial and endothelial cells (46–48) and immune response cells (49, 50). Thus, the significant reduction of attachment to and persistence in THP-1 cells supports the importance of Δ*pgn_1547* in *P. gingivalis* infection of host cells.

The transcriptional profile of the wild type shows the downregulation of genes associated with propanoate metabolism compared to the mutant. Short-chain fatty acids such as propionate are potent attractants to neutrophils in the subgingival cavity (51). Moreover, propionate has mild anti-inflammatory activity (52). On the other hand, genes associated with DNA repair and recombination proteins were upregulated in the wild type. Invasion by *P. gingivalis* induces the formation of reactive oxygen species (ROS), which play a crucial role in host innate immune responses (53–55). A direct consequence of ROS production is DNA damage. *P. gingivalis* has developed mechanisms to avoid this damage that would cause cell death (56, 57). Thus, the PG_1457 mutant does not seem to induce a robust immune response compared to the wild type.

Response of the THP-1 cells was also specific to the strain that infected them. At the initial response (2 hours), genes that have been associated with inflammation, bone loss, and susceptibility to periodontitis, such as FOSB and PLCG2, suppressors of cytokine signaling, and PTGS2, involved in prostaglandin biosynthesis (58–60), were upregulated in cells infected with the wild type. Moreover, PLCG2 is also involved in osteoclast differentiation and NF-κB signaling (61). Zhang et al. (62) showed lower levels of PTGS2 present in inflamed tissues in chronic periodontitis. Neutrophil extracellular trap formation (NET) was also upregulated in the wild type. In particular, genes H4C8 and H4C9, both synthesizing histones, were upregulated in the wild type compared to the mutant. Histones are the most abundant proteins in NETs and have bactericidal properties, both against Gram-positive and Gram-negative bacteria (63). These observations agree that the mutant does not mount a robust immune response compared to the wild type, as seen in the virulence experiments. At 6 hours, only interferon-inducible proteins were upregulated in the wild type. These genes, OAS2, MX1, and MX2, have been linked to the response of immunological pathways in rheumatoid arthritis and periodontitis (64).

The PGN_1547 mutant significantly decreased IL-6 and IL-1α and chemokines MIP-2α (CXCL2) and CXCL1 production, as well as IL-10 and IL8 at 6 hours. Although we did not see significant differences in TNF-α, we did observe a trend in reduced elicitation of TNF-α from THP-1 cells from the Δ*pgn_1547* mutant. Deregulation of TNF-α has been implicated in the pathogenesis of periodontitis (65). A similar finding was observed for IL-8. IL-8 is one of the principal mediators of the inflammatory response and a potent chemoattractant of neutrophils. IL-8 production decreased in the *pgn_1547* mutant. IL-8 attracts leukocytes to the site of infection, leading to neutrophil infiltration, which, if not controlled, may culminate in host tissue damage (66). Therefore, the downregulation of IL-8 production is vital in preventing chronic inflammation and tissue destruction caused by an influx of neutrophils indicated by the PGN_1547 mutant. One striking finding of this work is that the downregulation of IL-8 by the PGN_1547 mutant appears to be linked to the invasive ability of the Δ*pgn_1547* mutant. Previous studies have shown the involvement of a TonB-dependent receptor (RagB), lipopolysaccharide (LPS), and fimbria-dependent activation of IL-8 in primary human monocytes (67). The presence of low levels of IL-8, IL-6, and TNF-α in the PGN_1547 mutant may help decrease microbial fitness in the PGN_1547 mutant model. Similar results relating to less IL-8, IL-6, and IL-1α were reported when stimulated with the mutant biofilms compared to wild-type ones (68). Unexpectedly, we did not find significant changes in all cytokine and chemokines that were measured at the protein level using Luminex, yet trends were observed with several mediators suggesting that gene expression was different in response of THP-1 cells to wild-type *P. gingivalis* and the Δ*pgn_1547* mutant.

TLR-mediated recognition of bacterial components is an effective mechanism for rapidly defending against pathogens. Moreover, the interaction between pathogen components and the TLRs has implications beyond immediate nonspecific protection, as the activation may also be necessary for bridging innate and adaptive immune responses (69). We show that the purified PGN_1547 proteins are potent TLR2 agonists that can induce a solid proinflammatory cytokine response. We used the HEK-Blue hTLR cell assays that supported the idea that the PGN_1547 protein is sensed predominantly by TLR2. Notably, the PGN_1547 protein appeared to be a more potent TLR2 activator than TLR4, possibly corresponding to Δ PGN_1547 mutant’s lesser ability to induce cytokine production in THP-1 cells. TLR2 is one of the most promiscuous TLRs, mainly due to its heterodimerizing characteristics with either TLR1 or TLR6 upon interacting with a ligand. In addition, TLR4 recognizes bacterial LPS, while TLR2, along with TLR1 or TLR6, recognizes many pathogen-associated molecular pattern molecules (PAMPs), including lipoproteins and peptidoglycans, lipoteichoic acids, zymosan, mannan, and GPI-mucin (70). Several *P. gingivalis* proteins, such as FimA, signal through TLR2, while cytokine production by cells cultured with live *P. gingivalis* appears to signal through TLR2 and TLR4 (71).

In conclusion, we have identified the hypothetical protein PGN_1547 as a new virulence factor, probably acting as an adhesin whose levels of mRNAs are controlled by the type I CRISPR-Cas system of *P. gingivalis*.

## MATERIALS AND METHODS

### Bacterial growth conditions

Strains and plasmids utilized in this study are listed in Table S1. *P. gingivalis* ATCC 33277 was cultured anaerobically at 37°C. Brain heart infusion (BHI)-blood agar plates were used for cell maintenance, supplemented with 5 µg/mL hemin and 1 µg/mL menadione (vitamin K). Liquid cultures were made in tryptic soy broth (TSB) (BD, Becton, Dickinson, and Co.), supplemented with 1 µg/mL menadione and 5 µg/mL hemin. Erythromycin was used at a concentration of 10 µg/mL. Doubling time was calculated using the cal.double.time.curve.fit.py script from https://github.com/huoww07/calulate_bacteria_doubling_time. Differences between slopes were tested using the R package lsmeans to examine the ANOVA *P*-values.

### Construction of a PGN_1547 knockout strain of *Porphyromonas gingivalis*

To construct a PGN_1547 knockout strain of *P. gingivalis*, we replaced the entire gene with an erythromycin resistance cassette. First, we designed a plasmid (pUC19) carrying the erythromycin resistance cassette (ermF gene from pVA2198), flanked by a 1-kb region upstream and downstream of PGN_1547. The NEBuilder HiFi DNA assembly kit was used for this construct. Subsequently, the construct (the erythromycin cassette and its flanking regions) was PCR amplified using Pfu polymerase (Fermentas), following the manufacturer’s protocol.

The amplified fragments were purified using an EZNA gel extraction kit (Omega) and used for electroporation of *P. gingivalis* electrocompetent cells. Electrocompetent cells grew *P. gingivalis* ATCC33277 in tryptic soy broth supplemented with hemin and vitamin K to an optical density at 600 nm (OD_600_) of 0.6–0.7. After centrifugation, the cells were washed twice in ice-cold electroporation buffer (10% glycerol, 1 mM MgCl2) and resuspended in a minimal electroporation buffer. Electroporation was performed by adding different amounts of the purified DNA fragment to 100 µL of *P. gingivalis* competent cells. Tryptic soy broth blood agar plates supplemented with hemin, vitamin K, and 10 µg/mL erythromycin were used for the mutant selection. The plates were incubated anaerobically at 37°C for 9 days, and the resulting colonies were streaked on new plates to obtain single colonies.

Then, the PGN_1547 and CRISPR-30-36 gene knockout were verified using colony PCR with primers specific to the erythromycin cassette. The amplified products were confirmed by sequencing. The correct gene knockout strains were further grown in liquid media. Glycerol and dimethyl sulfoxide (DMSO) stocks were prepared and stored at −80°C. A list of all primers used in this study is provided in Table S2.

### *Galleria mellonella* infection model

The *Galleria mellonella* infection model was used for the experiments. Insects in the final instar larval stages were purchased from Vanderhorst, Inc. (St. Marys, OH, USA). Upon arrival, healthy larvae were separated from dead larvae and randomly assigned to different groups. Larvae weighing between 200 and 300 mg without any signs of melanization were selected. Each group consisted of 15 larvae, and seven groups were used for the infection. Infection was performed by injecting 5 µL aliquots of bacterial inoculum into each larva’s hemocoel through the last left proleg using a Hamilton syringe. Three groups received the PGN_1547 mutant, three received the wild-type *P. gingivalis*, and three control groups were included. The control groups consisted of THSB plus Δ*pgn*_1547 mutant heat killed (10 min at 75°C), tryptic soy broth medium alone, and TSB plus *P. gingivalis* wild type heat killed (10 min at 75°C). Similar groups were designed for the CRISPR-30-36 mutants. After injection, the larvae were incubated in the dark at 37°C. Melanization and survival of the larvae were recorded at specific time intervals. Larvae were considered dead if they showed no movement in response to touch. Survival analysis was performed using Kaplan-Meier killing curves, and the log-rank test was used to assess differences in survival. A *P*-value of ≤0.05 was considered significant. Data analysis was conducted using the “survival” and “survminer” packages in R. The experiments were repeated independently three times with similar results

### THP-1 cell culture

Human monocytes THP-1 (ATCC, TIB-202) were cultured in a 5% CO_2_ incubator at 37°C in RPMI 1640 and supplemented with L-glutamine (2 mM), heat-inactivated fetal bovine serum (10%), penicillin/streptomycin (1%), sodium pyruvate (1 mM), HEPES (10 mM), glucose (4.5 mg/mL), sodium bicarbonate (1.5 mg/mL), and 2-mercaptoethanol (0.05 mM; Sigma-Aldrich). The cells were standardized at 5 × 10^5^ cells/mL density and treated with 100 ng/mL phorbol 12-myristate 13-acetate (PMA; Sigma-Aldrich) to induce differentiation into a macrophage-like state. Later, 1 mL of THP-1 cell suspension was added to each well of 24-well cell culture plates. After 48 hours, the antibiotic-free medium replaced the cell culture medium, and cells received bacterial challenge.

### Bacterial infection experiments

Bacterial infection experiments were conducted as follows: *P. gingivalis* wild type and PGN_547 mutant strains were harvested from the BHI broth culture using centrifugation. The bacteria were washed three times with RPMI 1640 medium and adjusted to an optical density of 660 (OD_660_) of 1.0, corresponding to approximately 1 × 10^9^ CFU/mL. The bacterial cells were then added to PMA-activated THP-1 cells at a multiplicity of infection of 100 in an antibiotic-free medium. After 2 and 6 hours of infection, cell culture supernatant and RNA samples were harvested. The supernatant was collected to measure cytokine expression, while RNA was extracted for RT-qPCR analysis or RNA-seq. Before sample collection, the cultures were washed twice with 1 mL of PBS.

### Assessment of cytokine and chemokine production

The TNF-α, IL-1α, IL-6, IL-8, IL-10, and RANTES levels were determined using Milliplex multiplex assays (EMD, Millipore). Data were obtained on a MAGPIX multiplex reader system running xPONENT 3.1 software (Luminex) and examined using a five-parameter logistic spline-curve fitting method and Milliplex Analyst V5.1 software (Vigene Tech). Statistical differences were evaluated by two-way analysis of variance using the “means” package in R, applying a false discovery rate value of <0.05 for multiple-comparison corrections. Three independent experiments were performed.

### RNA extraction and library construction

Cells were washed three times in 1 mL of PBS. From THP-1 infection experiments, the total RNA of bacteria from inside THP-1 cells was extracted by using a *mir*Vana RNA isolation kit (Life Technologies). For 1 min, samples were bead-beaten in the *mir*Vana lysis buffer at maximum speed with 300 µL of 0.1 mm diethylpyrocarbonate-treated zirconia-silica beads (BioSpec Products). The manufacturer’s instructions were followed after the lysis steps.

Sequencing was performed at the Interdisciplinary Center for Biotechnology Research at the University of Florida using a HiSeq 2500 machine. First, rRNAs were removed from total RNA by Illumina Ribo-Zero Gold rRNA Removal Kit following the manufacturer’s protocol and eluted into 10 µL of EB buffer. Following the manufacturer’s recommendations, the RNA-seq library is processed using NEBNext Ultra Directional RNA Library Prep Kit for Illumina (NEB, USA). A total of 5 µL of depleted RNA mix and 5 µL of first-strand synthesis reaction mix (NEBNext First-strand Synthesis Reaction Buffer [5×] and NEBNext Random primers) were fragmented by heating at 94°C for the desired time. This step is followed by first-strand cDNA synthesis using reverse transcriptase and oligo dT primers. Synthesis of ds cDNA is performed using the second strand master mix provided in the kit, followed by end-repair and adaptor ligation. Finally, the library is enriched (each library has a unique barcode, each primer has a common adaptor sequence, which was added in the previous adaptor ligation step, and a unique overhang index unique to each sample) by a certain number of cycles of amplification and purified by Agencourt AMPure beads (Beckman Coulter, catalog #A63881). Finally, individual libraries were pooled with equimolar and sequenced by Illumina HiSeq 3000 2 × 100 cycles run (Illumina Inc., CA, USA).

### Illumina instrument run

Barcoded libraries were sized on the Agilent 2200 TapeStation to prepare for sequencing. Quantitation was done by QUBIT and qPCR (Kapa Biosystems, catalog number KK4824). Individual samples were pooled equimolarly at 2.5 nM. This “working pool” was used as input in the HiSeq3000 instrument sample preparation protocol (Illumina Material # 20015630, Document # 15066496 v04, January 2017). Typically, a 250 pM library concentration was used for clustering on the cBOT, resulting in an optimum clustering density at which the percentage of clusters passing filters was 65%–75%. Six RNA-seq barcoded libraries were pooled for sequencing in multiplex on a single flow cell lane, using a 2 × 100 cycles (paired-end) configuration. Such sequencing configuration was achieved by pooling the reagents from 150 cycles and 50 cycles of Illumina HiSeq3000 SBS kits. A typical sequencing run in the HiSeq3000 produced >300 million reads from each end per lane with a Q30 ≥ 90%. For RNA-seq, 50 million reads per end per sample provided sufficient depth for transcriptome analysis. The sequencing run was performed at NextGen of the Interdisciplinary Center for Biotechnology Research (University of Florida).

### Host-bacteria transcriptome analysis

Our study used the PATRIC annotation for genome ID 431947.7 of *P. gingivalis* sequences 33277 (25). Low-quality sequences were removed from the query files using Trimmomatic (72). Cleaned data were aligned against *the P. gingivalis* ATCC 33277 genome database using STAR aligner (73). Human sequences were aligned against genome release 33 (GRCh38.p14) in RefSeq assembly accession GCF_000001405.40_GRCh38.p14. Read counts from the BAM files were obtained using featureCounts (74).

Differential expression analysis was performed using DESeq2 (75). Enrichment of KEGG pathways was performed using the R package pathfindR (76). pathfindR identifies active subnetworks in a protein-protein interaction network using the list of differentially expressed genes obtained in the experiment comparing two groups of samples.

### Construction and purification of recombinant PGN_1547 protein

Construction and purification of recombinant 6×His-tagged PGN_1547 protein were performed as follows. The coding sequence (810 bp) of PGN_1547 protein without the signal peptide was cloned into the multiple cloning site (MCS-1) of the vector pET28b in-frame with the N-terminal hexahistidine residues using NcoI-HF and HindIII-HF restriction sites. The resulting expression plasmid construct was confirmed by restriction digestion analysis and direct sequencing with the T7 terminator and T7 promoter primers. Chemically competent *Escherichia coli* cells grown in Luria-Bertani broth supplemented with 50 µg/mL kanamycin were transformed with the plasmid. Induction was performed at 37°C with 0.5 mM IPTG when the OD at 600 nm reached 0.5, and cells were harvested by centrifugation after 4 hours of induction. The cell pellet was stored at −80°C until further use.

The harvested cells were resuspended in a lysis buffer containing 300 mM NaCl, 10 mM imidazole, and 50 mM NaH_2_PO_4_, supplemented with Complete EDTA-Free Protease Inhibitor tablets for the purification of the signal peptide-less 6×His-tagged recombinant PGN_1547 protein. The ratio of cell wet weight to buffer volume was maintained between 1:1 and 1:4, and 1 µL DNase solution (1 mg/mL) per milliliter of cell suspension was added to prevent viscosity problems. The cells were then subjected to French press at the recommended pressure (7,000–10,000 psi), and the lysate was collected. Cell debris was removed by ultracentrifugation at 4°C for 30 min at 45,000 rpm using a 45Ti rotor (Beckman).

The supernatant was loaded onto a pre-equilibrated 1 mL Ni-NTA column (Bio-Rad Laboratories, CA, USA) at 1 mL/min flow rate. SDS-PAGE analyzed unbound material. The column was washed with 40 mL of buffer containing 20 mM imidazole (pH 8.0), 300 mM NaCl, and 50 mM NaH_2_PO_4_. The bound protein was eluted using an elution buffer containing 300 mM NaCl, 250 mM imidazole (pH 8.0), and 50 mM NaH_2_PO_4_. Elution fractions were collected, pooled, and concentrated using an Amicon Ultra concentrator (Millipore) with a 10 kDa cut-off filter. The homogeneity of the protein was confirmed by Coomassie Blue staining of a 12% SDS-PAGE gel. The purified protein was dialyzed overnight at 4°C using HEPES dialysis buffer (500 mM NaCl, 2.5% glycerol, 10 mM HEPES pH 7.5) with a 3 kDa cut-off membrane.

### HEK-Blue cell cultivation and stimulation

HEK-Blue cell cultivation and stimulation were carried out as follows. Initially, HEK 293 cells (InvivoGen, San Diego, CA, USA) were cultured in DMEM with 10% fetal bovine serum (vol/vol), penicillin (100 U/mL), streptomycin (100 µg/mL), and Normocin (100 µg/mL) (InvivoGen, San Diego, CA, USA). After the second passage, 1× HEK-Blue selection (InvivoGen, San Diego, CA, USA) was added to the growth medium for TLR2 and TLR4 cells. Cell cultures were refreshed using PBS without centrifugation when the bottles reached 80% confluence. The cells were maintained at 37°C, 5% CO_2_, and appropriate humidity.

On the day of stimulation, the cells were detached using PBS, counted, and resuspended at a concentration of 140,000 cells/mL for hTLR4 and 280,000 cells/mL for hTLR2 in HEK-Blue Detection (InvivoGen, San Diego, CA, USA). Subsequently, 180 µL of the cell suspension was added to each well of a 96-well plate to be stimulated with 20 µL of purified PGN_1547 protein. The SEAP co-transfection with genes in HEK 293 cells allowed for the colorimetric reaction, which developed over time and was measured at 655 nm absorbance.

### Attachment of *P. gingivalis* wild-type strain and Δ*pgn_1547* mutant to human monocytes THP-1

Human monocytes THP-1 were grown as described previously and prepared for attachment assays by dilution to 5 × 10^5^ viable cells/mL in RPMI 1640. THP-1 cells were transferred into a fresh medium containing 100 ng/mL phorbol 12-myristate 13-acetate (Sigma-Aldrich) to induce differentiation into a macrophage-like state. Later, 1 mL of THP-1 cells was added to each well of 24-well cell culture plates. An antibiotic-free medium replaced the cell culture medium after 48 hours of incubation, and cells were used in challenge studies.

*P. gingivalis* wild-type strain and Δ*pgn_1547* mutant were harvested after 48 hours of growth on blood agar plates, washed twice, and resuspended in PBS. Bacteria were diluted to approximately 10^7^ cells/mL in RPMI 1640 without antibiotics, and 50 µL aliquots were added to each well containing THP-1 cells. The number of bacteria attached to the THP-1 cells after 120 min of incubation was expressed as the percentage of the number of bacteria added per monolayer. The results are from at least three experiments.

### Survival assay invasion/interaction

Cell culture plates were removed from the incubator, and media were aspirated and washed three times with anaerobic PBS. One milliliter of RPMI 1640 was added to the wild-type strain and Δ*pgn_1547* mutant and incubated for 60 min. After incubation, cells were washed three times with 1 mL PBS to remove unbound bacteria and were scraped from the bottom of each well with a cell scraper. The cells were collected from each well, lysed, serial dilutions performed, and spotted onto plates. Incubation was performed under anaerobic conditions, and colony-forming units were counted. This approach revealed the number of bacteria that both attached and were internalized within THP-1 cells.

### Survival of internalized bacteria

To determine only those bacteria that were able to survive intracellularly in THP-1 cells, we performed an antibiotic protection assay. The same protocol for “Survival assay invasion/interaction” (plating) was used except for the supplemented antibiotic (300 µg/mL of gentamicin and 400 µg/mL of metronidazole) to kill the bacteria attached to the THP-1 cells. The enumeration of bacteria at this stage was only internalized bacteria.

### Statistical analysis

All data are presented as the mean ± SD and evaluated by ANOVA (SPSS v.10.0). Differences between groups were considered significant at *P* < 0.05.

## Data Availability

RNA-seq data sets used in the paper can be accessed at NCBI at the BioProject ID PRJNA1037128.

## SUPPLEMENTAL MATERIAL

Fig. S1 msystems.01231-23-s0001.pdf Doubling time calculations. Calculations were performed using the scripts at https://github.com/huoww07/calulate_bacteria_doubling_timehttps://github.com/huoww07/calulate_bacteria_doubling_time.

Fig. S2 msystems.01231-23-s0002.pdf Effect of the PGN_1547 deletion on gene expression of *P. gingivalis*.

Fig. S3 msystems.01231-23-s0003.pdf Expression levels of genes analyzed by Luminex.

Table S1 msystems.01231-23-s0004.pdf Strains, plasmids, and primers used in this study.

Table S2 msystems.01231-23-s0005.pdf Differentially expressed genes in THP-1 cells when infected with *P. gingivalis* wild-type compared to the Δ*pgn_1547* mutant.

Table S3 msystems.01231-23-s0006.pdf Differentially expressed genes in *P. gingivalis* wild-type compared to the Δ*pgn_1547* mutant when infecting THP-1 cells.

ASM does not own the copyrights to Supplemental Material that may be linked to, or accessed through, an article. The authors have granted ASM a non-exclusive, world-wide license to publish the Supplemental Material files. Please contact the corresponding author directly for reuse.
