# Erratum for Irfan et al., “A *Porphyromonas gingivalis* hypothetical protein controlled by the type I-B CRISPR-Cas system is a novel adhesin important in virulence”

**DOI:** 10.1128/msystems.00295-25

**Published:** 2025-04-08

**Authors:** Muhammad Irfan, Jose Solbiati, Ana Duran-Pinedo, Fernanda Godoy Rocha, Frank C. Gibson, Jorge Frias-Lopez

## ERRATUM

Volume 9, no. 3, e01231-23, 2024, https://doi.org/10.1128/msystems.01231-23. The title should appear as given in this Erratum, and “type I-C CRISPR-Cas” should read “type I-B CRISPR-Cas” throughout. The nomenclature had changed prior to publication, but we were not aware of the modification at that time.

