# Supplementary material for: A Porphyromonas gingivalis hypothetical protein controlled by the type I-C CRISPR-Cas system is a novel adhesin important in virulence
Source: mSystems. 2024 Feb 7;9(3):e01231-23. doi: 10.1128/msystems.01231-23 (PMC10949514; doi:10.1128/msystems.01231-23)
Supplement: Fig. S1 — Doubling time calculations. Calculations were performed using the scripts at https://github.com/huoww07/calulate_bacteria_doubling_timehttps://github.com/huoww07/calulate_bacteria_doubling_time. [file msystems.01231-23-s0001.pdf]

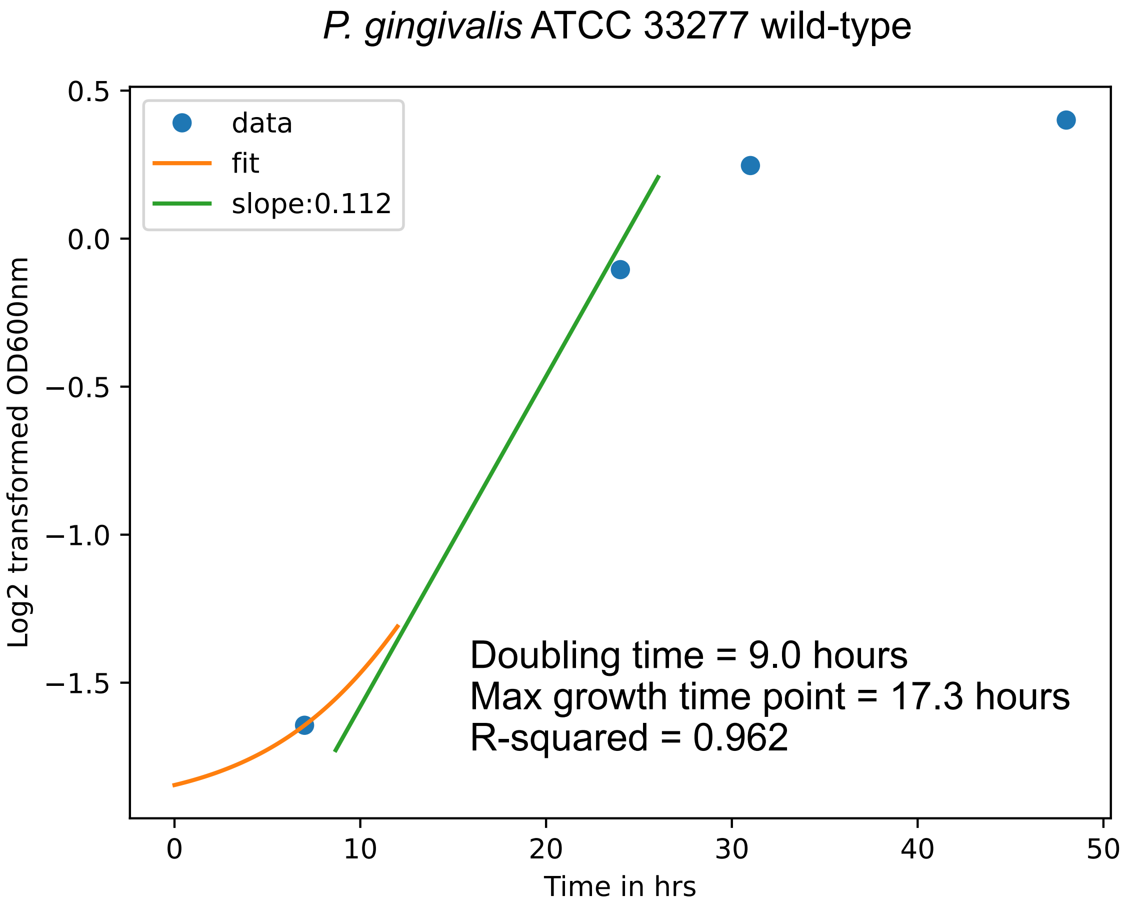


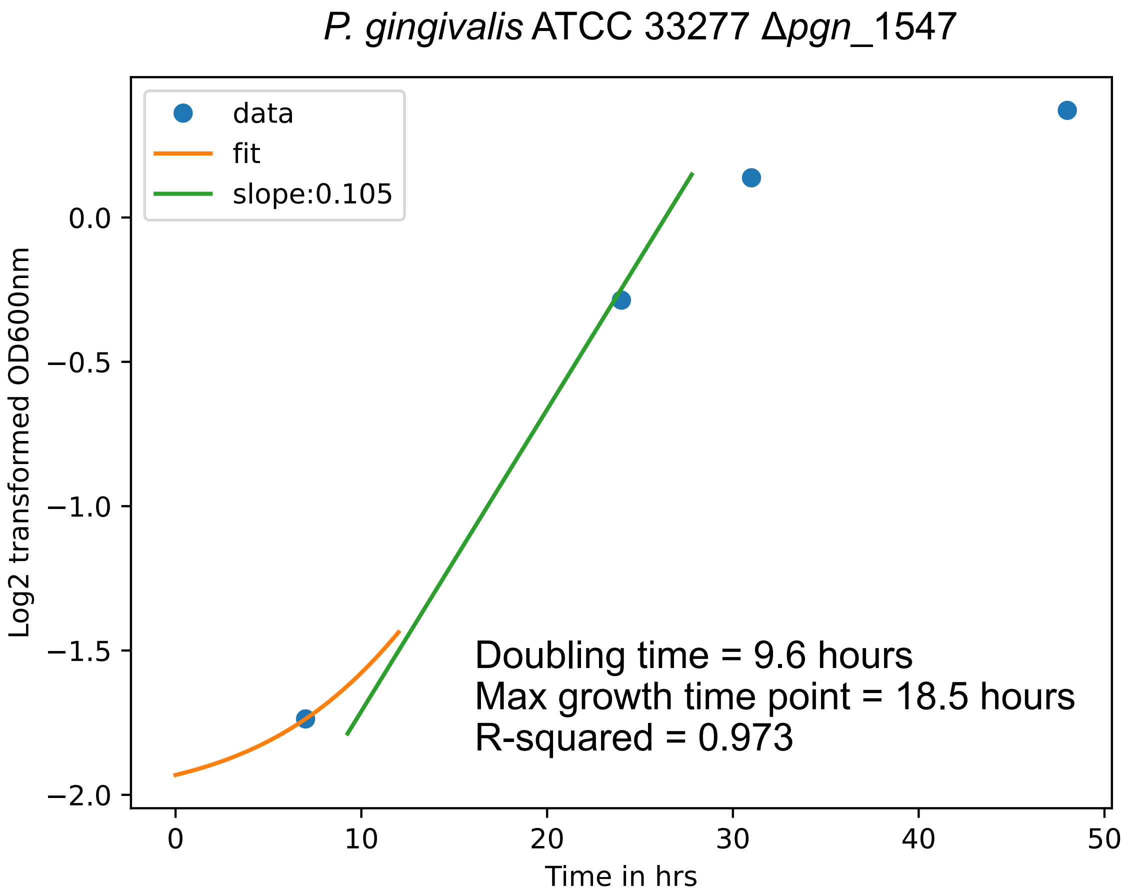


**FIG S1. Doubling time calculations.** Calculations were performed using the scripts at https://github.com/huoww07/calulate_bacteria_doubling_time.
