# Supplementary material for: A Porphyromonas gingivalis hypothetical protein controlled by the type I-C CRISPR-Cas system is a novel adhesin important in virulence
Source: mSystems. 2024 Feb 7;9(3):e01231-23. doi: 10.1128/msystems.01231-23 (PMC10949514; doi:10.1128/msystems.01231-23)
Supplement: Fig. S2 — Effect of the PGN_1547 deletion on gene expression of P. gingivalis. [file msystems.01231-23-s0002.pdf]

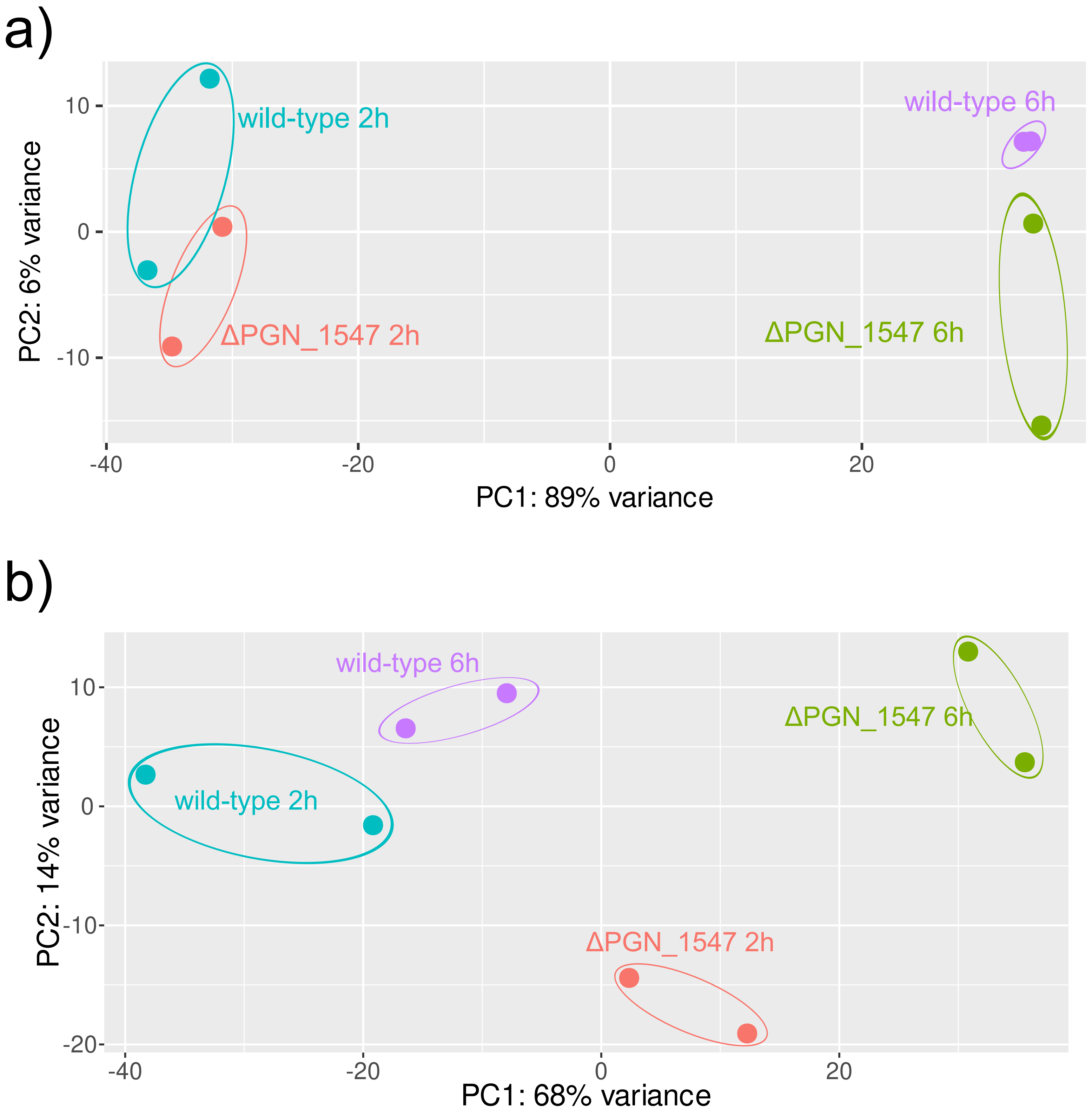

FIG S2. Effect of the PGN_1547 deletion on gene expression of *P. gingivalis.*
Transcriptomic analysis was performed on infected THP-1 cells.
Principal component analysis (PCA) of the wild-type and deletion mutants.
The transcriptomes from the different biological replicates for each condition are encircled.
a) THP-1 cell transcriptomes
b) *P. gingivalis* transcriptomes.
The analysis was performed at 2 and 6 hours.
