# Supplementary material for: A Porphyromonas gingivalis hypothetical protein controlled by the type I-C CRISPR-Cas system is a novel adhesin important in virulence
Source: mSystems. 2024 Feb 7;9(3):e01231-23. doi: 10.1128/msystems.01231-23 (PMC10949514; doi:10.1128/msystems.01231-23)
Supplement: Fig. S3 — Expression levels of genes analyzed by Luminex. [file msystems.01231-23-s0003.pdf]

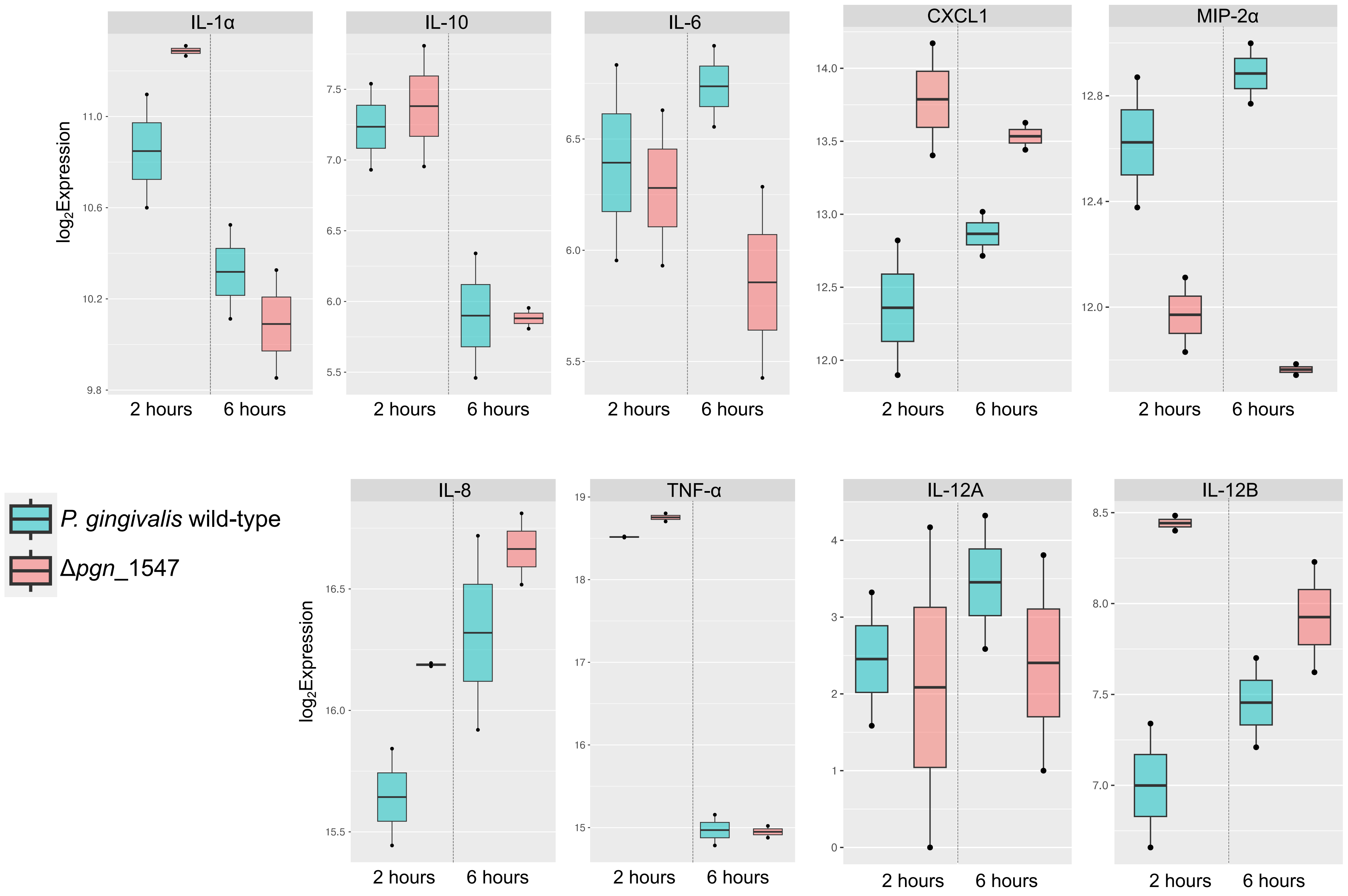

FIG S3. Expression levels of genes analyzed by Luminex.
Changes are represented in log2 of the mRNA counts for the different genes.
