# Supplementary material for: A Porphyromonas gingivalis hypothetical protein controlled by the type I-C CRISPR-Cas system is a novel adhesin important in virulence
Source: mSystems. 2024 Feb 7;9(3):e01231-23. doi: 10.1128/msystems.01231-23 (PMC10949514; doi:10.1128/msystems.01231-23)
Supplement: Table S1 — Strains, plasmids, and primers used in this study. [file msystems.01231-23-s0004.pdf]

Table S1. Strains, plasmids and primers used in this study.

**Name**	**Details**	**Sources**
***E. coli* strains**		
DH5α	used for cloning	NEB
BL21(DE3)	used for protein expression/purification	NEB
***Porphyromonas gingivalis* strains**		
Porphyromonas gingivalis ATCC 33277	Wild type strain of *P. gingivalis*	
Porphyromonas gingivalis ATCC 33277 Δ*pgn_1547* mutant	Mutant lacking PGN_1547 gene	This study
**Plasmids**		
pUC19	For making knockout construct of Δ*pgn_1547*	This study
pET28b	pET-28b derivative for expressing 6His-PGN_1547 protein	This study

Table S1. Strains, plasmids and primers used in this study.

Primer	5'-3' Sequence	Use
Before 1547-F	ctatgaccatgattacgccaGCAATTCCGTTCCTTCGG	For amplification of before fragment of PGN_1547
Before 1547 P-R	cgggggtaccAATTGACTGTTTTTAAGTTGATGATTC	For amplification of before fragment of PGN_1547
EMY Promotor-F	acagtcaattGGTACCCCCGATAGCTTC	For amplification of erythromycin cassette with promotor for knockout PGN_1547
EMY R	gtggaaccgcCTACGAAGGATGAAATTTTTCAGG	For amplification of erythromycin cassette with promotor for knockout PGN_1547
After 1547-F	tccttcgtagGCGGTTCCACATCTTAAG	For amplification of after fragment of PGN_1547
After 1547-R	ttgtaaaacgacggccagtgGATCCTTACGTCAGCTTG	For amplification of after fragment of PGN_1547
PGN_1547 KO Con-F	gcaattccgttccttcgggg	For amplification of entire knockout construct of PGN-1547
PGN_1547 Ko Con-R	gatccttacgtcagcttgttgg	For amplification of entire knockout construct of PGN-1547
PGN_1547 Exteral-F	CTTGACGTTCGCGATAGTCGA	For confirmation of clone in *Porphyromonas gingivalis knockout PGN_1547*
PGN_1547 Exteral-R	GGAAAGGATCCACGATTGATCG	For confirmation of clone in *Porphyromonas gingivalis*
M13/pUC Forward	CCC AGT CAC GAC GTT GTA AAA CG	For confirming construct in pUC19 plasmid
M13/pUC Reverse	AGC GGA TAA CAA TTT CAC ACA GG	For confirming construct in pUC19 plasmid
ErmF	CCGATAGCTTCCGCTATTGCTTTTTTGCTCATCGGT	Sequencing of knockout construct
ErmR	ACCGATGAGCAAAAAAGCAATAGCGGAAGCTATCGG	Sequencing of knockout construct
ckErm1-R	CGTAAATGTTCAACCAAAGCTGTG	Sequencing of knockout construct
qErmF	CCAACTGTCAAATCAGCCCTG	Sequencing of knockout construct
qErmR	CTTGGAGACAAACAAACAATT	Sequencing of knockout construct
T7 Promoter Primer	TAATACGACTCACTATAGGG	For confirming and sequencing construct in pET28b
T7 terminator	GCTAGTTATTGCTCAGCGG	For confirming and sequencing construct in pET28b
PGN_1547 F	tggtgctcgagtgcggccgcattacggttgcatactgaatgaagaa	Amplification of 1547 for purification in pET28b
1547 R Histag SP removed	actttaagaaggagatatacatgcatcatcaccaccaccatgacctccaaaaacgagtaaaagg	Amplification of 1547 for purification in pET28b
QPCR 1547 F	GCATCAGGCAGCATTGATATTC	QPCR primer for 1547
QPCR 1547 R	CGATGTTCAGGACACCTTTCT	QPCR primer for 1547
QPCR ragA-F	ACAGCCGTGCTACAGTAAAG	QPCR primer of internal control of *Porphyromonas gingivalis*
QPCR ragA-R	TAAACAACCGTCCGCCATAG	QPCR primer of internal control of *Porphyromonas gingivalis*
QOCR16s RNA F	CGGTTCCGTTCCGTCTTATT	QPCR primer of internal control of *Porphyromonas gingivalis*
QPCR 16s RNA R	CTCCATCTCCTCCTTGCTTTC	QPCR primer of internal control of *Porphyromonas gingivalis*
