# Supplementary material for: A Porphyromonas gingivalis hypothetical protein controlled by the type I-C CRISPR-Cas system is a novel adhesin important in virulence
Source: mSystems. 2024 Feb 7;9(3):e01231-23. doi: 10.1128/msystems.01231-23 (PMC10949514; doi:10.1128/msystems.01231-23)
Supplement: Table S2 — Differentially expressed genes in THP-1 cells when infected with P. gingivalis wild-type compared to the Δpgn_1547 mutant. [file msystems.01231-23-s0005.pdf]

Table S2. Results DESeq2 differential expression analysis. Results of THP-1 differentially expressed genes at 2 and 6 hours of infection with wild-type and PGN_1547 *P. gingivalis* ATCC 33277.

Differentially expressed genes 2 hours post-infection

Geneid	FoldChange	padj
LOC101929128	112	0.038
CSNK1A1L	31	0.042
LOC105372114	18	0.019
LOC101928160	13	0.007
TRU-TCA1-1	9	0.007
RPLP1P12	5	0.045
LOC128092249	4	0.005
TRH-GTG1-8	4	0.000
LOC101930434	4	0.001
TRH-GTG1-5	4	0.016
HNRNPA1P8	3	0.002
HNRNPA1P27	3	0.046
SCARNA7	3	0.009
TRE-CTC1-2	3	0.000
TRA-AGC1-1	3	0.001
TRE-CTC1-4	3	0.000
RNU1-3	3	0.000
TRA-AGC7-1	3	0.001
TRE-CTC1-3	3	0.000
RNU1-4	3	0.000
TRH-GTG1-7	3	0.007
TRE-CTC1-5	3	0.000
SCARNA17	3	0.017
RNVU1-18	3	0.000
MIR3609	3	0.004
RNY3P1	3	0.047
MUC12	3	0.012
RNVU1-7	3	0.000
RNU1-1	3	0.000
PTP4A2P1	3	0.034
RNVU1-14	3	0.011
SCARNA28	2	0.001
HSPA9P1	2	0.012

RN7SL4P	2	0.000
LOC124905574	2	0.001
TRH-GTG1-9	2	0.011
RNY3	2	0.007
TRR-CCT3-1	2	0.015
SCDP1	2	0.000
RN7SL674P	2	0.015
SNORD15B	2	0.017
TRR-ACG1-2	2	0.016
RN7SL396P	2	0.043
LY6G6C	2	0.003
RN7SL3	2	0.000
TRA-TGC7-1	2	0.001
RN7SKP48	2	0.000
PRNCR1	2	0.004
OLA1P1	2	0.001
RN7SKP175	2	0.005
RN7SKP80	2	0.000
SERHL2	2	0.000
MSNP1	2	0.005
ATP8B5P	2	0.009
HSP90AA4P	2	0.003
SNORA38	2	0.015
SNORA71A	2	0.030
TRD-GTC2-2	2	0.000
RN7SKP185	2	0.001
RN7SKP227	2	0.000
PABPC3	2	0.007
TRG-GCC1-4	2	0.012
NPR2	2	0.030
TRA-TGC5-1	2	0.012
TRD-GTC2-3	2	0.001
TRG-GCC1-3	2	0.012
HSP90AA2P	2	0.046
HSPA8P8	2	0.035
TRG-GCC1-2	2	0.012
LOC105370449	2	0.004
TRG-GCC1-1	2	0.017
EEF1A1P4	2	0.011

H4C13	2	0.000
MIR3652	2	0.000
SCARNA21	2	0.000
TUBAP2	2	0.011
SNORD3B-2	2	0.003
TRD-GTC2-4	2	0.004
LOC105379549	2	0.043
TRD-GTC2-5	2	0.010
SNORD17	2	0.003
HNRNPA1P7	2	0.010
FOSB	2	0.011
H4C5	2	0.000
MIR142HG	2	0.011
SCARNA2	2	0.025
CYB5D1	2	0.011
TRNP	2	0.015
SCARNA6	2	0.025
FRMD8	2	0.005
TNFSF14	2	0.038
EGR2	2	0.043
LOC124904106	1	0.011
PPP1R15A	1	0.002
LENG9	1	0.002
MIR23AHG	1	0.043
TAF15	1	0.015
SCARNA12	1	0.046
PTGS2	1	0.032
ZFP36	1	0.004
PLCG2	1	0.005
CSRNP1	1	0.019
BTBD19	1	0.020
SELENOT	-1	0.028
SELENOF	-2	0.007
SNORA105B	-2	0.049
GON7	-2	0.009
NDUFAF5	-2	0.039
PRH1-TAS2R14	-2	0.003
PRH1-PRR4	-2	0.007
PRH1	-2	0.001

LOC124904211	-2	0.043
SOX5-AS1	-3	0.027
LILRA4	-19	0.021
LOC124903511	-112	0.021

Differentially expressed genes 6 hours post-infection

Geneid	FoldChange	padj
SLC25A51P1	229	0.038
NLRP7	141	0.042
LOC107987463	77	0.019
MT2P1	11	0.007
NT5C1A	4	0.007
SRRM1P1	4	0.045
RNU5B-1	4	0.005
EEF1A1P22	4	0.000
PGK1P2	3	0.001
RN7SKP227	3	0.016
TRA-TGC7-1	3	0.002
WASF4P	3	0.046
TRA-TGC5-1	3	0.009
RNU1-2	3	0.000
RNU1-3	3	0.001
LOC124905574	3	0.000
RNU1-4	3	0.000
BCYRN1P1	3	0.001
RNVU1-18	3	0.000
RN7SKP48	3	0.000
BCYRN1	2	0.007
SCARNA7	2	0.000
RNU1-1	2	0.017
RNVU1-7	2	0.000
TRNE	2	0.004
ATP8	2	0.047
ND6	2	0.012
ND4L	2	0.000
ND2	2	0.000
TRNL1	2	0.034
LILRA2	2	0.011
MX1	2	0.001
OAS2	2	0.012
MX2	2	0.000
TRNI	2	0.001

HERC6	2	0.011
SP110	2	0.007
PARP9	1	0.015
LOC107983985	-145	0.000
GAPDHP14	-162	0.015
