# Supplementary material for: A Porphyromonas gingivalis hypothetical protein controlled by the type I-C CRISPR-Cas system is a novel adhesin important in virulence
Source: mSystems. 2024 Feb 7;9(3):e01231-23. doi: 10.1128/msystems.01231-23 (PMC10949514; doi:10.1128/msystems.01231-23)
Supplement: Table S3 — Differentially expressed genes in P. gingivalis wild-type compared to the Δpgn_1547 mutant when infecting THP-1 cells. [file msystems.01231-23-s0006.pdf]

Table S3. Results DESeq2 differential expression analysis. Results of *P. gingivalis* ATCC 33277 differentially expressed genes at 2 and 6 hours of infection with wild-type and PGN_1547 *P. gingivalis* ATCC 33277.

Differentially expressed genes 2 hours post-infection

Gene	PATRIC annotation	log2FoldChange	padj
PGN_1547	fig|431947.7.peg.1530	8	0.00
	fig|431947.7.peg.998	4	0.01
	fig|431947.7.peg.159	3	0.04
PGN_1161	fig|431947.7.peg.1153	3	0.01
PGN_0575	fig|431947.7.peg.568	2	0.00
PGN_2090	fig|431947.7.peg.2055	2	0.02
PGN_1823	fig|431947.7.peg.1794	2	0.00
	fig|431947.7.peg.429	2	0.00
PGN_1416	fig|431947.7.peg.1403	2	0.00
PGN_1517	fig|431947.7.peg.1500	2	0.00
	fig|431947.7.peg.1402	2	0.00
PGN_1519	fig|431947.7.peg.1502	2	0.00
PGN_1827	fig|431947.7.peg.1798	2	0.00
PGN_1132	fig|431947.7.peg.1123	2	0.00
PGN_1300	fig|431947.7.peg.1290	2	0.00
PGN_0577	fig|431947.7.peg.571	2	0.02
PGN_0102	fig|431947.7.peg.95	2	0.00
PGN_1518	fig|431947.7.peg.1501	2	0.00
PGN_0420	fig|431947.7.peg.419	2	0.00
PGN_1274	fig|431947.7.peg.1265	2	0.00
PGN_1619	fig|431947.7.peg.1597	2	0.04
PGN_1089	fig|431947.7.peg.1077	2	0.01
	fig|431947.7.peg.316	2	0.00
PGN_1378	fig|431947.7.peg.1366	2	0.00
PGN_1335	fig|431947.7.peg.1322	2	0.00
PGN_2082	fig|431947.7.peg.2046	2	0.00
PGN_1906	fig|431947.7.peg.1877	2	0.00
PGN_1411	fig|431947.7.peg.1398	2	0.00
PGN_1904	fig|431947.7.peg.1875	2	0.00
PGN_1408	fig|431947.7.peg.1396	2	0.00
PGN_1321	fig|431947.7.peg.1309	2	0.00
PGN_1403	fig|431947.7.peg.1392	2	0.00
PGN_1982	fig|431947.7.peg.1947	2	0.00

PGN_0218	fig|431947.7.peg.215	2	0.04
PGN_1006	fig|431947.7.peg.996	2	0.00
PGN_1208	fig|431947.7.peg.1202	2	0.00
PGN_1366	fig|431947.7.peg.1356	2	0.00
PGN_1798	fig|431947.7.peg.1773	2	0.01
	fig|431947.7.peg.1685	2	0.00
PGN_0909	fig|431947.7.peg.898	2	0.00
PGN_0900	fig|431947.7.peg.889	2	0.00
PGN_0773	fig|431947.7.peg.763	2	0.00
PGN_0944	fig|431947.7.peg.934	1	0.00
PGN_0879	fig|431947.7.peg.870	1	0.00
PGN_0341	fig|431947.7.peg.338	1	0.00
PGN_1920	fig|431947.7.peg.1891	1	0.00
PGN_0178	fig|431947.7.peg.172	1	0.01
PGN_0856	fig|431947.7.peg.846	1	0.05
PGN_1065	fig|431947.7.peg.1055	1	0.00
PGN_1988	fig|431947.7.peg.1954	1	0.00
PGN_0955	fig|431947.7.peg.945	1	0.00
PGN_0661	fig|431947.7.peg.652	1	0.00
PGN_1418	fig|431947.7.peg.1404	1	0.00
PGN_1118	fig|431947.7.peg.1106	1	0.01
PGN_0606	fig|431947.7.peg.602	1	0.00
PGN_0946	fig|431947.7.peg.936	1	0.00
PGN_1115	fig|431947.7.peg.1101	1	0.00
PGN_1275	fig|431947.7.peg.1266	1	0.00
PGN_0103	fig|431947.7.peg.96	1	0.00
PGN_2037	fig|431947.7.peg.2001	1	0.00
PGN_0210	fig|431947.7.peg.205	1	0.01
PGN_0748	fig|431947.7.peg.735	1	0.00
PGN_1060	fig|431947.7.peg.1050	1	0.00
PGN_1633	fig|431947.7.peg.1610	1	0.00
PGN_0999	fig|431947.7.peg.988	1	0.00
PGN_0464	fig|431947.7.peg.464	1	0.00
PGN_1402	fig|431947.7.peg.1391	1	0.00
PGN_1481	fig|431947.7.peg.1463	1	0.00
PGN_0389	fig|431947.7.peg.387	1	0.00
PGN_1064	fig|431947.7.peg.1054	1	0.00
PGN_1057	fig|431947.7.peg.1047	1	0.00
PGN_2001	fig|431947.7.peg.1967	1	0.00

PGN_0687	fig|431947.7.peg.677	1	0.00
PGN_0267	fig|431947.7.peg.266	1	0.01
PGN_1733	fig|431947.7.peg.1709	1	0.00
PGN_1715	fig|431947.7.peg.1690	1	0.00
PGN_1032	fig|431947.7.peg.1022	1	0.00
PGN_2065	fig|431947.7.peg.2029	1	0.00
PGN_2081	fig|431947.7.peg.2045	1	0.00
PGN_1763	fig|431947.7.peg.1740	1	0.00
PGN_0916	fig|431947.7.peg.905	1	0.00
PGN_0477	fig|431947.7.peg.477	1	0.00
PGN_1407	fig|431947.7.peg.1395	1	0.00
PGN_0370	fig|431947.7.peg.369	1	0.00
PGN_1622	fig|431947.7.peg.1599	1	0.00
PGN_1454	fig|431947.7.peg.1435	1	0.01
PGN_0943	fig|431947.7.peg.933	1	0.00
PGN_1880	fig|431947.7.peg.1851	1	0.00
PGN_0631	fig|431947.7.peg.625	1	0.00
PGN_1992	fig|431947.7.peg.1958	1	0.00
PGN_0180	fig|431947.7.peg.174	1	0.00
PGN_1245	fig|431947.7.peg.1237	1	0.00
PGN_1074	fig|431947.7.peg.1064	1	0.01
PGN_0348	fig|431947.7.peg.345	1	0.00
PGN_1521	fig|431947.7.peg.1504	1	0.01
PGN_0614	fig|431947.7.peg.610	1	0.00
PGN_0967	fig|431947.7.peg.958	1	0.00
PGN_1412	fig|431947.7.peg.1399	1	0.00
PGN_1187	fig|431947.7.peg.1180	1	0.00
PGN_1009	fig|431947.7.peg.999	1	0.00
PGN_0285	fig|431947.7.peg.283	1	0.00
PGN_0216	fig|431947.7.peg.212	1	0.00
PGN_2048	fig|431947.7.peg.2013	1	0.00
PGN_0718	fig|431947.7.peg.706	1	0.03
PGN_1388	fig|431947.7.peg.1376	1	0.00
PGN_1747	fig|431947.7.peg.1723	1	0.00
PGN_1126	fig|431947.7.peg.1116	1	0.00
PGN_1364	fig|431947.7.peg.1353	1	0.00
PGN_1409	fig|431947.7.peg.1397	1	0.00
PGN_0707	fig|431947.7.peg.697	1	0.00
PGN_1725	fig|431947.7.peg.1700	1	0.00

PGN_1119	fig|431947.7.peg.1107	1	0.00
PGN_1746	fig|431947.7.peg.1722	1	0.00
PGN_1716	fig|431947.7.peg.1691	1	0.00
PGN_1052	fig|431947.7.peg.1042	1	0.00
PGN_0476	fig|431947.7.peg.476	1	0.00
PGN_0418	fig|431947.7.peg.417	1	0.00
PGN_1268	fig|431947.7.peg.1259	1	0.00
PGN_0595	fig|431947.7.peg.591	1	0.03
PGN_1383	fig|431947.7.peg.1370	1	0.00
PGN_0439	fig|431947.7.peg.439	1	0.00
PGN_0380	fig|431947.7.peg.378	1	0.01
PGN_1795	fig|431947.7.peg.1768	1	0.01
PGN_1539	fig|431947.7.peg.1523	1	0.01
PGN_0980	fig|431947.7.peg.969	1	0.01
	fig|431947.7.peg.906	1	0.00
PGN_0220	fig|431947.7.peg.217	1	0.00
PGN_0300	fig|431947.7.peg.298	1	0.00
PGN_0241	fig|431947.7.peg.239	1	0.00
PGN_1516	fig|431947.7.peg.1499	1	0.04
PGN_1522	fig|431947.7.peg.1505	1	0.00
PGN_0376	fig|431947.7.peg.374	1	0.02
PGN_1428	fig|431947.7.peg.1413	1	0.01
PGN_1226	fig|431947.7.peg.1220	1	0.03
PGN_0798	fig|431947.7.peg.787	1	0.00
PGN_0583	fig|431947.7.peg.577	1	0.01
PGN_0837	fig|431947.7.peg.826	1	0.00
PGN_1969	fig|431947.7.peg.1935	1	0.00
PGN_1893	fig|431947.7.peg.1864	1	0.00
PGN_1916	fig|431947.7.peg.1887	1	0.01
PGN_0310	fig|431947.7.peg.307	1	0.01
PGN_0302	fig|431947.7.peg.300	1	0.00
PGN_1090	fig|431947.7.peg.1078	1	0.00
PGN_0162	fig|431947.7.peg.157	1	0.00
PGN_1638	fig|431947.7.peg.1615	1	0.01
	fig|431947.7.peg.1388	1	0.00
PGN_1833	fig|431947.7.peg.1804	1	0.00
	fig|431947.7.peg.1407	1	0.02
PGN_0350	fig|431947.7.peg.348	1	0.01
PGN_1491	fig|431947.7.peg.1473	1	0.02

PGN_0427	fig|431947.7.peg.426	1	0.00
PGN_1269	fig|431947.7.peg.1260	1	0.00
PGN_0429	fig|431947.7.peg.428	1	0.00
PGN_1053	fig|431947.7.peg.1043	1	0.01
PGN_0400	fig|431947.7.peg.396	1	0.00
PGN_0282	fig|431947.7.peg.280	1	0.01
PGN_1419	fig|431947.7.peg.1405	1	0.00
PGN_0349	fig|431947.7.peg.346	1	0.00
PGN_1579	fig|431947.7.peg.1560	1	0.00
	fig|431947.7.peg.1389	1	0.00
PGN_0428	fig|431947.7.peg.427	1	0.00
PGN_0753	fig|431947.7.peg.742	1	0.01
	fig|431947.7.peg.211	1	0.00
PGN_0248	fig|431947.7.peg.246	1	0.02
PGN_1986	fig|431947.7.peg.1951	1	0.02
PGN_0894	fig|431947.7.peg.885	1	0.00
PGN_1617	fig|431947.7.peg.1595	1	0.01
PGN_0838	fig|431947.7.peg.828	1	0.00
	fig|431947.7.peg.2012	1	0.00
PGN_2073	fig|431947.7.peg.2038	1	0.00
	fig|431947.7.peg.438	1	0.00
PGN_0836	fig|431947.7.peg.825	1	0.00
PGN_1800	fig|431947.7.peg.1774	1	0.00
PGN_1169	fig|431947.7.peg.1161	1	0.00
PGN_1564	fig|431947.7.peg.1544	1	0.00
PGN_1556	fig|431947.7.peg.1539	1	0.00
PGN_0933	fig|431947.7.peg.922	1	0.01
PGN_2070	fig|431947.7.peg.2035	1	0.00
PGN_1641	fig|431947.7.peg.1618	1	0.03
PGN_0466	fig|431947.7.peg.466	1	0.02
PGN_0270	fig|431947.7.peg.269	1	0.03
PGN_0284	fig|431947.7.peg.282	1	0.01
PGN_1081	fig|431947.7.peg.1072	1	0.00
PGN_1014	fig|431947.7.peg.1004	1	0.00
PGN_0735	fig|431947.7.peg.721	1	0.01
PGN_1039	fig|431947.7.peg.1030	1	0.04
PGN_1987	fig|431947.7.peg.1953	1	0.01
PGN_1260	fig|431947.7.peg.1250	1	0.00
PGN_0391	fig|431947.7.peg.389	1	0.00

PGN_1796	fig|431947.7.peg.1769	1	0.01
PGN_1314	fig|431947.7.peg.1302	1	0.01
PGN_1401	fig|431947.7.peg.1390	1	0.00
PGN_1640	fig|431947.7.peg.1617	1	0.04
PGN_0055	fig|431947.7.peg.48	1	0.00
PGN_0004	fig|431947.7.peg.4	1	0.03
PGN_0535	fig|431947.7.peg.531	1	0.01
PGN_1991	fig|431947.7.peg.1957	1	0.00
PGN_1252	fig|431947.7.peg.1242	1	0.00
PGN_1136	fig|431947.7.peg.1127	1	0.05
PGN_1993	fig|431947.7.peg.1959	1	0.00
PGN_1745	fig|431947.7.peg.1721	1	0.00
PGN_0557	fig|431947.7.peg.551	1	0.01
PGN_1164	fig|431947.7.peg.1156	1	0.00
PGN_1246	fig|431947.7.peg.1238	1	0.01
PGN_1994	fig|431947.7.peg.1960	1	0.01
PGN_0618	fig|431947.7.peg.613	1	0.00
PGN_1286	fig|431947.7.peg.1278	1	0.00
PGN_1714	fig|431947.7.peg.1689	1	0.01
PGN_0107	fig|431947.7.peg.101	1	0.01
PGN_0065	fig|431947.7.peg.58	1	0.00
PGN_2033	fig|431947.7.peg.1997	1	0.04
PGN_0041	fig|431947.7.peg.36	1	0.00
PGN_1565	fig|431947.7.peg.1545	1	0.00
PGN_2016	fig|431947.7.peg.1980	1	0.01
PGN_0917	fig|431947.7.peg.907	1	0.01
PGN_0889	fig|431947.7.peg.880	1	0.03
PGN_0648	fig|431947.7.peg.642	1	0.02
PGN_1737	fig|431947.7.peg.1714	1	0.01
PGN_0992	fig|431947.7.peg.981	1	0.04
PGN_1598	fig|431947.7.peg.1579	1	0.01
PGN_0733	fig|431947.7.peg.719	1	0.00
PGN_0219	fig|431947.7.peg.216	1	0.02
PGN_1159	fig|431947.7.peg.1151	1	0.00
PGN_0473	fig|431947.7.peg.473	1	0.00
PGN_1700	fig|431947.7.peg.1674	1	0.00
PGN_1452	fig|431947.7.peg.1434	1	0.03
	fig|431947.7.peg.1598	1	0.03
PGN_0264	fig|431947.7.peg.263	1	0.04

PGN_0795	fig|431947.7.peg.785	1	0.01
PGN_1717	fig|431947.7.peg.1692	1	0.01
PGN_1570	fig|431947.7.peg.1551	1	0.03
PGN_0686	fig|431947.7.peg.676	1	0.02
PGN_1387	fig|431947.7.peg.1375	1	0.03
PGN_1043	fig|431947.7.peg.1034	1	0.04
PGN_0532	fig|431947.7.peg.528	1	0.00
PGN_0531	fig|431947.7.peg.527	1	0.00
PGN_1562	fig|431947.7.peg.1543	1	0.02
PGN_1358	fig|431947.7.peg.1347	1	0.02
PGN_1513	fig|431947.7.peg.1496	1	0.00
PGN_1834	fig|431947.7.peg.1805	1	0.03
PGN_0890	fig|431947.7.peg.881	1	0.01
PGN_1129	fig|431947.7.peg.1119	1	0.03
PGN_0262	fig|431947.7.peg.261	1	0.04
PGN_1561	fig|431947.7.peg.1542	1	0.01
PGN_0351	fig|431947.7.peg.349	1	0.01
PGN_0375	fig|431947.7.peg.373	1	0.01
PGN_1094	fig|431947.7.peg.1082	1	0.03
PGN_1533	fig|431947.7.peg.1517	1	0.01
PGN_1168	fig|431947.7.peg.1160	1	0.02
PGN_0736	fig|431947.7.peg.722	1	0.03
PGN_0221	fig|431947.7.peg.218	1	0.02
PGN_1749	fig|431947.7.peg.1725	1	0.03
PGN_0211	fig|431947.7.peg.207	1	0.02
PGN_2003	fig|431947.7.peg.1968	1	0.02
PGN_1441	fig|431947.7.peg.1424	1	0.03
PGN_1309	fig|431947.7.peg.1297	1	0.04
PGN_2079	fig|431947.7.peg.2043	1	0.03
PGN_0728	fig|431947.7.peg.716	1	0.05
PGN_1614	fig|431947.7.peg.1592	1	0.04
PGN_0009	fig|431947.7.peg.9	1	0.03
PGN_0463	fig|431947.7.peg.463	1	0.04
PGN_1979	fig|431947.7.peg.1943	1	0.03
PGN_1399	fig|431947.7.peg.1387	1	0.03
PGN_1498	fig|431947.7.peg.1481	1	0.01
PGN_1773	fig|431947.7.peg.1747	1	0.01
PGN_1771	fig|431947.7.peg.1745	1	0.04
PGN_1878	fig|431947.7.peg.1849	1	0.03

PGN_1437	fig|431947.7.peg.1422	1	0.05
PGN_1536	fig|431947.7.peg.1520	1	0.02
PGN_1693	fig|431947.7.peg.1668	1	0.02
PGN_0722	fig|431947.7.peg.710	1	0.03
PGN_1689	fig|431947.7.peg.1664	1	0.02
PGN_1019	fig|431947.7.peg.1009	1	0.02
PGN_1685	fig|431947.7.peg.1661	1	0.02
PGN_0123	fig|431947.7.peg.117	1	0.03
PGN_1670	fig|431947.7.peg.1645	1	0.05
PGN_1786	fig|431947.7.peg.1759	1	0.01
PGN_1451	fig|431947.7.peg.1433	1	0.04
PGN_1955	fig|431947.7.peg.1922	0	0.03
PGN_0173	fig|431947.7.peg.168	0	0.02
PGN_0568	fig|431947.7.peg.560	-1	0.05
PGN_0705	fig|431947.7.peg.695	-1	0.03
PGN_1756	fig|431947.7.peg.1732	-1	0.05
PGN_0710	fig|431947.7.peg.700	-1	0.04
PGN_1601	fig|431947.7.peg.1582	-1	0.04
PGN_0138	fig|431947.7.peg.132	-1	0.03
PGN_0415	fig|431947.7.peg.414	-1	0.04
PGN_0799	fig|431947.7.peg.788	-1	0.03
PGN_1743	fig|431947.7.peg.1719	-1	0.03
PGN_0962	fig|431947.7.peg.953	-1	0.01
PGN_0952	fig|431947.7.peg.942	-1	0.01
PGN_1702	fig|431947.7.peg.1676	-1	0.01
PGN_1377	fig|431947.7.peg.1365	-1	0.04
PGN_0409	fig|431947.7.peg.407	-1	0.03
PGN_1457	fig|431947.7.peg.1439	-1	0.05
PGN_0876	fig|431947.7.peg.867	-1	0.01
PGN_1356	fig|431947.7.peg.1344	-1	0.03
PGN_0208	fig|431947.7.peg.203	-1	0.04
PGN_0639	fig|431947.7.peg.633	-1	0.01
PGN_1117	fig|431947.7.peg.1104	-1	0.03
PGN_1354	fig|431947.7.peg.1343	-1	0.01
PGN_1303	fig|431947.7.peg.1293	-1	0.04
PGN_1581	fig|431947.7.peg.1562	-1	0.03
PGN_1091	fig|431947.7.peg.1079	-1	0.00
PGN_1964	fig|431947.7.peg.1929	-1	0.01
PGN_1108	fig|431947.7.peg.1095	-1	0.01

PGN_0732	fig|431947.7.peg.718	-1	0.03
PGN_0977	fig|431947.7.peg.968	-1	0.04
PGN_1489	fig|431947.7.peg.1471	-1	0.01
PGN_1367	fig|431947.7.peg.1357	-1	0.03
PGN_1088	fig|431947.7.peg.1076	-1	0.04
PGN_1317	fig|431947.7.peg.1305	-1	0.04
PGN_1186	fig|431947.7.peg.1179	-1	0.00
PGN_0079	fig|431947.7.peg.71	-1	0.00
PGN_1466	fig|431947.7.peg.1447	-1	0.03
PGN_0906	fig|431947.7.peg.896	-1	0.01
PGN_1096	fig|431947.7.peg.1084	-1	0.04
PGN_0891	fig|431947.7.peg.882	-1	0.02
PGN_0546	fig|431947.7.peg.542	-1	0.02
PGN_1166	fig|431947.7.peg.1158	-1	0.05
PGN_0082	fig|431947.7.peg.75	-1	0.01
PGN_1503	fig|431947.7.peg.1487	-1	0.01
PGN_1941	fig|431947.7.peg.1910	-1	0.05
PGN_1691	fig|431947.7.peg.1666	-1	0.02
PGN_1654	fig|431947.7.peg.1631	-1	0.01
PGN_1914	fig|431947.7.peg.1885	-1	0.00
PGN_0200	fig|431947.7.peg.195	-1	0.01
PGN_1179	fig|431947.7.peg.1171	-1	0.00
PGN_1264	fig|431947.7.peg.1254	-1	0.03
PGN_0188	fig|431947.7.peg.183	-1	0.03
PGN_0664	fig|431947.7.peg.655	-1	0.01
PGN_0604	fig|431947.7.peg.600	-1	0.00
PGN_0295	fig|431947.7.peg.292	-1	0.04
PGN_0510	fig|431947.7.peg.506	-1	0.03
PGN_1341	fig|431947.7.peg.1327	-1	0.01
PGN_1220	fig|431947.7.peg.1214	-1	0.00
PGN_1486	fig|431947.7.peg.1468	-1	0.01
PGN_0068	fig|431947.7.peg.61	-1	0.00
PGN_0743	fig|431947.7.peg.730	-1	0.00
PGN_0033	fig|431947.7.peg.30	-1	0.00
PGN_1576	fig|431947.7.peg.1557	-1	0.02
PGN_2021	fig|431947.7.peg.1985	-1	0.03
PGN_1775	fig|431947.7.peg.1749	-1	0.04
PGN_0830	fig|431947.7.peg.818	-1	0.02
PGN_0622	fig|431947.7.peg.616	-1	0.02

PGN_0042	fig|431947.7.peg.37	-1	0.00
PGN_0377	fig|431947.7.peg.375	-1	0.02
PGN_1655	fig|431947.7.peg.1632	-1	0.00
PGN_0573	fig|431947.7.peg.565	-1	0.01
PGN_1739	fig|431947.7.peg.1716	-1	0.02
PGN_0667	fig|431947.7.peg.658	-1	0.04
PGN_0135	fig|431947.7.peg.129	-1	0.02
PGN_1318	fig|431947.7.peg.1306	-1	0.00
PGN_2061	fig|431947.7.peg.2026	-1	0.05
	fig|431947.7.peg.408	-1	0.01
PGN_2054	fig|431947.7.peg.2019	-1	0.04
	fig|431947.7.peg.1479	-1	0.02
PGN_0187	fig|431947.7.peg.182	-1	0.00
PGN_0044	fig|431947.7.peg.39	-1	0.02
PGN_0293	fig|431947.7.peg.290	-1	0.00
PGN_0831	fig|431947.7.peg.819	-1	0.01
PGN_0379	fig|431947.7.peg.377	-1	0.04
PGN_1323	fig|431947.7.peg.1310	-1	0.00
PGN_1962	fig|431947.7.peg.1927	-1	0.02
PGN_0791	fig|431947.7.peg.781	-1	0.05
PGN_0340	fig|431947.7.peg.337	-1	0.05
PGN_0223	fig|431947.7.peg.221	-1	0.02
PGN_0357	fig|431947.7.peg.357	-1	0.00
PGN_0410	fig|431947.7.peg.409	-1	0.00
PGN_0371	fig|431947.7.peg.370	-1	0.01
PGN_1932	fig|431947.7.peg.1902	-1	0.04
PGN_0078	fig|431947.7.peg.70	-1	0.00
PGN_1181	fig|431947.7.peg.1173	-1	0.00
PGN_0880	fig|431947.7.peg.871	-1	0.00
PGN_1575	fig|431947.7.peg.1556	-1	0.00
PGN_0765	fig|431947.7.peg.755	-1	0.04
PGN_0361	fig|431947.7.peg.361	-1	0.02
PGN_2008	fig|431947.7.peg.1973	-1	0.04
PGN_1357	fig|431947.7.peg.1345	-1	0.04
PGN_0416	fig|431947.7.peg.415	-1	0.00
PGN_1848	fig|431947.7.peg.1818	-1	0.00
PGN_1271	fig|431947.7.peg.1262	-1	0.03
PGN_1496	fig|431947.7.peg.1478	-1	0.01
PGN_0607	fig|431947.7.peg.603	-1	0.00

PGN_0803	fig|431947.7.peg.792	-1	0.00
PGN_0975	fig|431947.7.peg.966	-1	0.03
PGN_0709	fig|431947.7.peg.699	-1	0.00
PGN_0806	fig|431947.7.peg.795	-1	0.00
PGN_0726	fig|431947.7.peg.714	-1	0.00
PGN_0858	fig|431947.7.peg.848	-1	0.02
PGN_1505	fig|431947.7.peg.1489	-1	0.05
PGN_0299	fig|431947.7.peg.297	-1	0.00
PGN_1320	fig|431947.7.peg.1308	-1	0.00
PGN_0671	fig|431947.7.peg.662	-1	0.00
PGN_1282	fig|431947.7.peg.1274	-1	0.01
PGN_0491	fig|431947.7.peg.489	-1	0.00
PGN_1857	fig|431947.7.peg.1827	-1	0.05
PGN_1577	fig|431947.7.peg.1558	-1	0.00
PGN_0411	fig|431947.7.peg.410	-1	0.00
PGN_0291	fig|431947.7.peg.289	-1	0.01
PGN_0750	fig|431947.7.peg.738	-1	0.01
PGN_0931	fig|431947.7.peg.921	-1	0.03
PGN_0534	fig|431947.7.peg.530	-1	0.03
PGN_0190	fig|431947.7.peg.185	-1	0.00
PGN_0119	fig|431947.7.peg.113	-1	0.01
PGN_1178	fig|431947.7.peg.1170	-1	0.00
PGN_1469	fig|431947.7.peg.1449	-1	0.00
PGN_0358	fig|431947.7.peg.358	-1	0.00
PGN_0294	fig|431947.7.peg.291	-1	0.00
PGN_1488	fig|431947.7.peg.1470	-1	0.01
PGN_1808	fig|431947.7.peg.1782	-1	0.02
PGN_1963	fig|431947.7.peg.1928	-1	0.00
PGN_1574	fig|431947.7.peg.1555	-1	0.00
PGN_1852	fig|431947.7.peg.1822	-1	0.03
PGN_1475	fig|431947.7.peg.1456	-1	0.03
PGN_1673	fig|431947.7.peg.1648	-1	0.00
PGN_0076	fig|431947.7.peg.69	-1	0.00
PGN_1871	fig|431947.7.peg.1841	-1	0.01
PGN_2007	fig|431947.7.peg.1972	-1	0.02
PGN_1723	fig|431947.7.peg.1698	-1	0.03
PGN_0741	fig|431947.7.peg.728	-1	0.00
PGN_0154	fig|431947.7.peg.149	-1	0.00
PGN_1339	fig|431947.7.peg.1325	-1	0.02

PGN_1230	fig|431947.7.peg.1224	-1	0.00
PGN_0509	fig|431947.7.peg.505	-1	0.00
PGN_0543	fig|431947.7.peg.539	-1	0.00
PGN_0404	fig|431947.7.peg.401	-1	0.02
PGN_0550	fig|431947.7.peg.546	-1	0.00
PGN_1858	fig|431947.7.peg.1828	-1	0.02
PGN_1853	fig|431947.7.peg.1823	-1	0.00
PGN_0412	fig|431947.7.peg.411	-1	0.00
PGN_0804	fig|431947.7.peg.793	-1	0.00
PGN_0289	fig|431947.7.peg.287	-1	0.00
PGN_0094	fig|431947.7.peg.87	-1	0.00
PGN_1851	fig|431947.7.peg.1821	-1	0.00
PGN_0675	fig|431947.7.peg.666	-1	0.01
PGN_1049	fig|431947.7.peg.1040	-1	0.00
PGN_0290	fig|431947.7.peg.288	-1	0.03
PGN_0963	fig|431947.7.peg.954	-1	0.00
PGN_0965	fig|431947.7.peg.956	-1	0.00
PGN_0665	fig|431947.7.peg.656	-1	0.00
PGN_0059	fig|431947.7.peg.52	-1	0.00
	fig|431947.7.peg.894	-1	0.05
	fig|431947.7.peg.1178	-1	0.01
PGN_0359	fig|431947.7.peg.359	-1	0.00
PGN_0818	fig|431947.7.peg.807	-1	0.00
PGN_1865	fig|431947.7.peg.1835	-1	0.02
PGN_0805	fig|431947.7.peg.794	-1	0.00
PGN_1724	fig|431947.7.peg.1699	-1	0.01
PGN_2060	fig|431947.7.peg.2025	-1	0.00
PGN_0571	fig|431947.7.peg.563	-1	0.03
PGN_0226	fig|431947.7.peg.224	-1	0.02
PGN_0746	fig|431947.7.peg.733	-1	0.05
PGN_0610	fig|431947.7.peg.606	-1	0.00
PGN_1817	fig|431947.7.peg.1790	-1	0.01
PGN_0080	fig|431947.7.peg.72	-1	0.00
PGN_0829	fig|431947.7.peg.817	-1	0.01
PGN_1185	fig|431947.7.peg.1177	-1	0.00
PGN_1867	fig|431947.7.peg.1837	-1	0.00
PGN_0757	fig|431947.7.peg.747	-1	0.04
PGN_1313	fig|431947.7.peg.1301	-1	0.02
PGN_0279	fig|431947.7.peg.278	-1	0.00

PGN_1302	fig|431947.7.peg.1292	-1	0.01
PGN_1461	fig|431947.7.peg.1443	-1	0.01
PGN_0760	fig|431947.7.peg.750	-1	0.01
PGN_1242	fig|431947.7.peg.1233	-1	0.01
PGN_0191	fig|431947.7.peg.186	-1	0.00
PGN_1849	fig|431947.7.peg.1819	-1	0.01
	fig|431947.7.peg.926	-1	0.00
PGN_1854	fig|431947.7.peg.1824	-1	0.00
PGN_0091	fig|431947.7.peg.84	-1	0.02
PGN_0964	fig|431947.7.peg.955	-1	0.00
PGN_1219	fig|431947.7.peg.1213	-1	0.00
PGN_1573	fig|431947.7.peg.1554	-1	0.00
PGN_1928	fig|431947.7.peg.1898	-1	0.02
PGN_0515	fig|431947.7.peg.511	-1	0.00
PGN_0612	fig|431947.7.peg.608	-1	0.00
PGN_1902	fig|431947.7.peg.1873	-1	0.00
PGN_0490	fig|431947.7.peg.488	-1	0.00
PGN_0274	fig|431947.7.peg.273	-1	0.00
PGN_0902	fig|431947.7.peg.891	-1	0.00
PGN_0724	fig|431947.7.peg.712	-1	0.00
PGN_1239	fig|431947.7.peg.1231	-1	0.02
PGN_0725	fig|431947.7.peg.713	-1	0.00
PGN_1572	fig|431947.7.peg.1553	-1	0.00
PGN_1930	fig|431947.7.peg.1900	-1	0.00
PGN_0668	fig|431947.7.peg.659	-1	0.00
PGN_1458	fig|431947.7.peg.1440	-1	0.00
PGN_1856	fig|431947.7.peg.1826	-1	0.00
PGN_1929	fig|431947.7.peg.1899	-1	0.01
PGN_1021	fig|431947.7.peg.1011	-1	0.02
PGN_2080	fig|431947.7.peg.2044	-1	0.00
PGN_1777	fig|431947.7.peg.1751	-1	0.00
PGN_1218	fig|431947.7.peg.1212	-1	0.00
PGN_1111	fig|431947.7.peg.1097	-1	0.00
PGN_0904	fig|431947.7.peg.893	-1	0.00
PGN_1840	fig|431947.7.peg.1810	-1	0.00
PGN_0758	fig|431947.7.peg.748	-1	0.01
PGN_0514	fig|431947.7.peg.510	-1	0.03
PGN_0827	fig|431947.7.peg.815	-1	0.00
PGN_1855	fig|431947.7.peg.1825	-1	0.00

PGN_1866	fig|431947.7.peg.1836	-1	0.00
PGN_0117	fig|431947.7.peg.111	-1	0.00
PGN_1240	fig|431947.7.peg.1232	-1	0.00
PGN_0549	fig|431947.7.peg.545	-1	0.00
PGN_0109	fig|431947.7.peg.103	-1	0.00
	fig|431947.7.peg.883	-1	0.01
PGN_0566	fig|431947.7.peg.558	-1	0.00
PGN_0287	fig|431947.7.peg.285	-1	0.00
	fig|431947.7.peg.73	-1	0.04
PGN_1847	fig|431947.7.peg.1817	-1	0.00
PGN_1868	fig|431947.7.peg.1838	-1	0.00
PGN_0723	fig|431947.7.peg.711	-1	0.00
PGN_1842	fig|431947.7.peg.1812	-1	0.00
PGN_1953	fig|431947.7.peg.1920	-1	0.00
PGN_0273	fig|431947.7.peg.272	-1	0.00
PGN_1764	fig|431947.7.peg.1741	-1	0.00
PGN_0742	fig|431947.7.peg.729	-1	0.00
PGN_0508	fig|431947.7.peg.504	-2	0.00
PGN_0693	fig|431947.7.peg.683	-2	0.00
PGN_0564	fig|431947.7.peg.556	-2	0.00
PGN_0691	fig|431947.7.peg.681	-2	0.00
PGN_0547	fig|431947.7.peg.543	-2	0.00
PGN_1080	fig|431947.7.peg.1071	-2	0.00
PGN_1859	fig|431947.7.peg.1829	-2	0.00
PGN_0118	fig|431947.7.peg.112	-2	0.00
PGN_1121	fig|431947.7.peg.1109	-2	0.03
PGN_0471	fig|431947.7.peg.471	-2	0.00
PGN_0288	fig|431947.7.peg.286	-2	0.00
PGN_0692	fig|431947.7.peg.682	-2	0.00
PGN_0611	fig|431947.7.peg.607	-2	0.00
PGN_1841	fig|431947.7.peg.1811	-2	0.00
PGN_0099	fig|431947.7.peg.92	-2	0.00
PGN_0110	fig|431947.7.peg.104	-2	0.00
PGN_1860	fig|431947.7.peg.1830	-2	0.00
PGN_0024	fig|431947.7.peg.22	-2	0.00
PGN_0623	fig|431947.7.peg.617	-2	0.00
PGN_0545	fig|431947.7.peg.541	-2	0.00
PGN_1462	fig|431947.7.peg.1444	-2	0.00
PGN_1843	fig|431947.7.peg.1813	-2	0.00

PGN_1844	fig|431947.7.peg.1814	-2	0.00
PGN_0939	fig|431947.7.peg.928	-2	0.00
PGN_0023	fig|431947.7.peg.21	-2	0.00
PGN_1846	fig|431947.7.peg.1816	-2	0.00
PGN_1845	fig|431947.7.peg.1815	-2	0.00
PGN_0548	fig|431947.7.peg.544	-2	0.00
	fig|431947.7.peg.727	-2	0.01
PGN_0224	fig|431947.7.peg.222	-2	0.00
	fig|431947.7.peg.1783	-2	0.00
PGN_2009	fig|431947.7.peg.1974	-2	0.02
PGN_0940	fig|431947.7.peg.929	-2	0.00
PGN_1692	fig|431947.7.peg.1667	-2	0.02
	fig|431947.7.peg.88	-2	0.00
PGN_1738	fig|431947.7.peg.1715	-3	0.00
	fig|431947.7.peg.1966	-3	0.00
PGN_0407	fig|431947.7.peg.405	-4	0.02

Differentially expressed genes 6 hours post-infection

Gene	PATRIC annotation	log2FoldChange	padj
PGN_1547	fig|431947.7.peg.1530	9	0.00
	fig|431947.7.peg.810	4	0.00
PGN_0536	fig|431947.7.peg.532	3	0.00
	fig|431947.7.peg.2034	3	0.05
PGN_1276	fig|431947.7.peg.1268	3	0.00
PGN_1077	fig|431947.7.peg.1068	3	0.00
PGN_0388	fig|431947.7.peg.386	3	0.00
PGN_1030	fig|431947.7.peg.1020	3	0.00
PGN_0601	fig|431947.7.peg.597	3	0.01
PGN_0753	fig|431947.7.peg.742	3	0.00
PGN_0752	fig|431947.7.peg.741	3	0.00
	fig|431947.7.peg.176	2	0.00
PGN_2090	fig|431947.7.peg.2055	2	0.00
PGN_0924	fig|431947.7.peg.914	2	0.00
PGN_0842	fig|431947.7.peg.832	2	0.01
PGN_0373	fig|431947.7.peg.371	2	0.00
PGN_1295	fig|431947.7.peg.1286	2	0.02
PGN_1161	fig|431947.7.peg.1153	2	0.01
	fig|431947.7.peg.1402	2	0.00
PGN_0008	fig|431947.7.peg.7	2	0.00
PGN_0784	fig|431947.7.peg.775	2	0.00
PGN_1232	fig|431947.7.peg.1225	2	0.00
PGN_1206	fig|431947.7.peg.1199	2	0.00
PGN_0493	fig|431947.7.peg.491	2	0.00
PGN_1115	fig|431947.7.peg.1101	2	0.00
PGN_1221	fig|431947.7.peg.1215	2	0.00
PGN_0845	fig|431947.7.peg.837	2	0.01
PGN_1132	fig|431947.7.peg.1123	2	0.00
PGN_0574	fig|431947.7.peg.567	2	0.00
PGN_0936	fig|431947.7.peg.925	2	0.00
PGN_1679	fig|431947.7.peg.1655	2	0.00
PGN_0661	fig|431947.7.peg.652	2	0.00
PGN_0954	fig|431947.7.peg.944	2	0.00
PGN_1682	fig|431947.7.peg.1659	2	0.00
PGN_2065	fig|431947.7.peg.2029	2	0.00
PGN_0846	fig|431947.7.peg.838	2	0.03

PGN_1420	fig|431947.7.peg.1406	2	0.00
PGN_1669	fig|431947.7.peg.1644	2	0.00
PGN_1416	fig|431947.7.peg.1403	2	0.00
PGN_2037	fig|431947.7.peg.2001	2	0.00
PGN_1321	fig|431947.7.peg.1309	2	0.00
PGN_0575	fig|431947.7.peg.568	2	0.00
PGN_0604	fig|431947.7.peg.600	2	0.00
PGN_1427	fig|431947.7.peg.1412	2	0.04
PGN_0644	fig|431947.7.peg.638	2	0.03
PGN_2074	fig|431947.7.peg.2039	2	0.00
PGN_0103	fig|431947.7.peg.96	2	0.00
PGN_1292	fig|431947.7.peg.1283	2	0.02
PGN_1118	fig|431947.7.peg.1106	2	0.00
PGN_0749	fig|431947.7.peg.737	2	0.01
PGN_0043	fig|431947.7.peg.38	2	0.00
PGN_1182	fig|431947.7.peg.1174	2	0.00
PGN_0569	fig|431947.7.peg.561	2	0.00
PGN_1275	fig|431947.7.peg.1266	2	0.00
PGN_0587	fig|431947.7.peg.582	2	0.01
PGN_1191	fig|431947.7.peg.1184	2	0.00
PGN_1433	fig|431947.7.peg.1418	2	0.00
PGN_0210	fig|431947.7.peg.205	2	0.00
PGN_0042	fig|431947.7.peg.37	2	0.00
PGN_1827	fig|431947.7.peg.1798	2	0.00
PGN_1260	fig|431947.7.peg.1250	2	0.03
PGN_0773	fig|431947.7.peg.763	2	0.00
PGN_0687	fig|431947.7.peg.677	2	0.00
PGN_0234	fig|431947.7.peg.231	2	0.00
PGN_1454	fig|431947.7.peg.1435	2	0.00
PGN_0603	fig|431947.7.peg.599	2	0.00
PGN_1408	fig|431947.7.peg.1396	2	0.00
PGN_1378	fig|431947.7.peg.1366	2	0.00
PGN_1926	fig|431947.7.peg.1896	2	0.00
PGN_0324	fig|431947.7.peg.322	2	0.00
PGN_0220	fig|431947.7.peg.217	2	0.00
PGN_0595	fig|431947.7.peg.591	2	0.00
PGN_1880	fig|431947.7.peg.1851	2	0.00
PGN_0577	fig|431947.7.peg.571	2	0.00
PGN_1522	fig|431947.7.peg.1505	2	0.00

PGN_0690	fig|431947.7.peg.680	2	0.00
PGN_0302	fig|431947.7.peg.300	2	0.00
PGN_0721	fig|431947.7.peg.709	2	0.00
PGN_0102	fig|431947.7.peg.95	2	0.00
PGN_1526	fig|431947.7.peg.1511	2	0.00
PGN_0722	fig|431947.7.peg.710	2	0.00
PGN_0660	fig|431947.7.peg.651	2	0.00
PGN_0219	fig|431947.7.peg.216	2	0.00
PGN_1335	fig|431947.7.peg.1322	2	0.00
PGN_0598	fig|431947.7.peg.594	2	0.00
PGN_0923	fig|431947.7.peg.913	2	0.01
PGN_0442	fig|431947.7.peg.442	2	0.00
PGN_0492	fig|431947.7.peg.490	2	0.00
PGN_0838	fig|431947.7.peg.828	2	0.00
PGN_0159	fig|431947.7.peg.153	2	0.00
PGN_0429	fig|431947.7.peg.428	2	0.00
PGN_0900	fig|431947.7.peg.889	2	0.00
PGN_0879	fig|431947.7.peg.870	2	0.00
PGN_0909	fig|431947.7.peg.898	2	0.00
PGN_1705	fig|431947.7.peg.1679	2	0.00
PGN_0380	fig|431947.7.peg.378	2	0.00
PGN_0890	fig|431947.7.peg.881	2	0.00
PGN_0944	fig|431947.7.peg.934	2	0.00
PGN_1186	fig|431947.7.peg.1179	2	0.00
PGN_0918	fig|431947.7.peg.908	2	0.00
PGN_1274	fig|431947.7.peg.1265	2	0.00
	fig|431947.7.peg.727	2	0.01
PGN_0588	fig|431947.7.peg.583	2	0.01
PGN_0837	fig|431947.7.peg.826	2	0.00
PGN_0720	fig|431947.7.peg.708	2	0.00
PGN_1412	fig|431947.7.peg.1399	2	0.00
PGN_0454	fig|431947.7.peg.454	1	0.00
PGN_0955	fig|431947.7.peg.945	1	0.00
PGN_0834	fig|431947.7.peg.823	1	0.02
PGN_1556	fig|431947.7.peg.1539	1	0.00
PGN_1490	fig|431947.7.peg.1472	1	0.00
PGN_1533	fig|431947.7.peg.1517	1	0.00
PGN_1704	fig|431947.7.peg.1678	1	0.00
PGN_1187	fig|431947.7.peg.1180	1	0.00

PGN_0185	fig|431947.7.peg.180	1	0.00
PGN_1411	fig|431947.7.peg.1398	1	0.00
PGN_1364	fig|431947.7.peg.1353	1	0.00
PGN_1733	fig|431947.7.peg.1709	1	0.00
PGN_1006	fig|431947.7.peg.996	1	0.00
PGN_1403	fig|431947.7.peg.1392	1	0.00
	fig|431947.7.peg.1407	1	0.00
PGN_1906	fig|431947.7.peg.1877	1	0.00
PGN_1681	fig|431947.7.peg.1658	1	0.05
PGN_0783	fig|431947.7.peg.774	1	0.00
PGN_1032	fig|431947.7.peg.1022	1	0.00
PGN_1419	fig|431947.7.peg.1405	1	0.00
PGN_0702	fig|431947.7.peg.692	1	0.00
PGN_1428	fig|431947.7.peg.1413	1	0.00
PGN_0593	fig|431947.7.peg.589	1	0.00
PGN_1904	fig|431947.7.peg.1875	1	0.00
	fig|431947.7.peg.502	1	0.03
PGN_1119	fig|431947.7.peg.1107	1	0.00
PGN_0420	fig|431947.7.peg.419	1	0.00
PGN_0050	fig|431947.7.peg.44	1	0.02
PGN_0160	fig|431947.7.peg.154	1	0.00
PGN_0561	fig|431947.7.peg.554	1	0.00
PGN_0065	fig|431947.7.peg.58	1	0.00
PGN_0285	fig|431947.7.peg.283	1	0.00
PGN_0599	fig|431947.7.peg.595	1	0.03
PGN_0971	fig|431947.7.peg.962	1	0.01
PGN_0349	fig|431947.7.peg.346	1	0.00
	fig|431947.7.peg.926	1	0.00
PGN_0606	fig|431947.7.peg.602	1	0.00
PGN_1935	fig|431947.7.peg.1905	1	0.00
PGN_0631	fig|431947.7.peg.625	1	0.00
PGN_1913	fig|431947.7.peg.1884	1	0.00
PGN_0594	fig|431947.7.peg.590	1	0.00
PGN_0718	fig|431947.7.peg.706	1	0.00
PGN_1366	fig|431947.7.peg.1356	1	0.00
PGN_2077	fig|431947.7.peg.2041	1	0.00
PGN_0748	fig|431947.7.peg.735	1	0.00
PGN_1129	fig|431947.7.peg.1119	1	0.00
PGN_1057	fig|431947.7.peg.1047	1	0.00

	fig|431947.7.peg.740	1	0.00
PGN_1836	fig|431947.7.peg.1806	1	0.05
PGN_0436	fig|431947.7.peg.436	1	0.04
PGN_0265	fig|431947.7.peg.264	1	0.00
PGN_0877	fig|431947.7.peg.868	1	0.00
PGN_1920	fig|431947.7.peg.1891	1	0.00
	fig|431947.7.peg.1685	1	0.00
PGN_2073	fig|431947.7.peg.2038	1	0.00
PGN_0946	fig|431947.7.peg.936	1	0.00
PGN_1245	fig|431947.7.peg.1237	1	0.05
PGN_2071	fig|431947.7.peg.2036	1	0.00
PGN_1065	fig|431947.7.peg.1055	1	0.00
PGN_1747	fig|431947.7.peg.1723	1	0.00
PGN_0922	fig|431947.7.peg.912	1	0.02
PGN_1409	fig|431947.7.peg.1397	1	0.00
PGN_0476	fig|431947.7.peg.476	1	0.00
PGN_0389	fig|431947.7.peg.387	1	0.00
PGN_1136	fig|431947.7.peg.1127	1	0.00
	fig|431947.7.peg.827	1	0.00
PGN_0430	fig|431947.7.peg.430	1	0.00
PGN_1060	fig|431947.7.peg.1050	1	0.00
PGN_0564	fig|431947.7.peg.556	1	0.00
PGN_0131	fig|431947.7.peg.125	1	0.01
PGN_0132	fig|431947.7.peg.126	1	0.00
PGN_0568	fig|431947.7.peg.560	1	0.00
PGN_1774	fig|431947.7.peg.1748	1	0.00
PGN_0348	fig|431947.7.peg.345	1	0.00
PGN_0999	fig|431947.7.peg.988	1	0.00
PGN_1316	fig|431947.7.peg.1304	1	0.00
PGN_0592	fig|431947.7.peg.588	1	0.05
PGN_0741	fig|431947.7.peg.728	1	0.00
	fig|431947.7.peg.429	1	0.00
PGN_0684	fig|431947.7.peg.674	1	0.00
PGN_1740	fig|431947.7.peg.1717	1	0.00
	fig|431947.7.peg.316	1	0.00
PGN_1758	fig|431947.7.peg.1735	1	0.00
PGN_0284	fig|431947.7.peg.282	1	0.00
PGN_1622	fig|431947.7.peg.1599	1	0.00
PGN_0933	fig|431947.7.peg.922	1	0.00

PGN_1028	fig|431947.7.peg.1018	1	0.00
PGN_1226	fig|431947.7.peg.1220	1	0.00
PGN_1383	fig|431947.7.peg.1370	1	0.00
PGN_0112	fig|431947.7.peg.105	1	0.01
PGN_1396	fig|431947.7.peg.1384	1	0.00
PGN_1521	fig|431947.7.peg.1504	1	0.00
PGN_1279	fig|431947.7.peg.1271	1	0.00
PGN_1189	fig|431947.7.peg.1182	1	0.00
	fig|431947.7.peg.351	1	0.05
PGN_1353	fig|431947.7.peg.1342	1	0.00
PGN_1767	fig|431947.7.peg.1742	1	0.00
PGN_1978	fig|431947.7.peg.1942	1	0.00
PGN_0795	fig|431947.7.peg.785	1	0.00
PGN_0967	fig|431947.7.peg.958	1	0.00
PGN_0466	fig|431947.7.peg.466	1	0.00
PGN_1064	fig|431947.7.peg.1054	1	0.00
PGN_1052	fig|431947.7.peg.1042	1	0.00
PGN_0464	fig|431947.7.peg.464	1	0.00
PGN_0004	fig|431947.7.peg.4	1	0.00
PGN_1670	fig|431947.7.peg.1645	1	0.00
PGN_1839	fig|431947.7.peg.1809	1	0.01
	fig|431947.7.peg.324	1	0.01
	fig|431947.7.peg.148	1	0.03
PGN_0637	fig|431947.7.peg.631	1	0.00
PGN_0943	fig|431947.7.peg.933	1	0.00
PGN_0326	fig|431947.7.peg.323	1	0.00
PGN_1988	fig|431947.7.peg.1954	1	0.00
PGN_1517	fig|431947.7.peg.1500	1	0.00
PGN_1994	fig|431947.7.peg.1960	1	0.00
PGN_1901	fig|431947.7.peg.1872	1	0.01
PGN_0427	fig|431947.7.peg.426	1	0.00
PGN_0264	fig|431947.7.peg.263	1	0.00
PGN_0341	fig|431947.7.peg.338	1	0.00
PGN_1070	fig|431947.7.peg.1060	1	0.01
PGN_0267	fig|431947.7.peg.266	1	0.00
PGN_1076	fig|431947.7.peg.1066	1	0.00
PGN_0707	fig|431947.7.peg.697	1	0.00
PGN_0966	fig|431947.7.peg.957	1	0.00
PGN_2070	fig|431947.7.peg.2035	1	0.00

PGN_1951	fig|431947.7.peg.1919	1	0.00
PGN_1912	fig|431947.7.peg.1883	1	0.00
PGN_1126	fig|431947.7.peg.1116	1	0.00
PGN_0618	fig|431947.7.peg.613	1	0.00
PGN_1309	fig|431947.7.peg.1297	1	0.00
PGN_1081	fig|431947.7.peg.1072	1	0.00
PGN_0836	fig|431947.7.peg.825	1	0.00
PGN_1995	fig|431947.7.peg.1961	1	0.00
PGN_1491	fig|431947.7.peg.1473	1	0.01
PGN_0370	fig|431947.7.peg.369	1	0.00
PGN_1993	fig|431947.7.peg.1959	1	0.00
PGN_0477	fig|431947.7.peg.477	1	0.00
PGN_1066	fig|431947.7.peg.1056	1	0.00
PGN_0735	fig|431947.7.peg.721	1	0.00
PGN_1300	fig|431947.7.peg.1290	1	0.00
PGN_1039	fig|431947.7.peg.1030	1	0.00
PGN_1987	fig|431947.7.peg.1953	1	0.00
PGN_0790	fig|431947.7.peg.780	1	0.00
PGN_1418	fig|431947.7.peg.1404	1	0.00
PGN_0375	fig|431947.7.peg.373	1	0.00
PGN_1680	fig|431947.7.peg.1657	1	0.01
	fig|431947.7.peg.1572	1	0.03
PGN_0657	fig|431947.7.peg.647	1	0.00
PGN_1135	fig|431947.7.peg.1126	1	0.00
PGN_1693	fig|431947.7.peg.1668	1	0.00
PGN_1208	fig|431947.7.peg.1202	1	0.00
PGN_0107	fig|431947.7.peg.101	1	0.00
PGN_1579	fig|431947.7.peg.1560	1	0.00
PGN_0970	fig|431947.7.peg.961	1	0.00
PGN_0585	fig|431947.7.peg.580	1	0.00
PGN_0729	fig|431947.7.peg.717	1	0.00
PGN_1687	fig|431947.7.peg.1662	1	0.00
PGN_1519	fig|431947.7.peg.1502	1	0.00
PGN_0423	fig|431947.7.peg.422	1	0.00
PGN_2016	fig|431947.7.peg.1980	1	0.00
	fig|431947.7.peg.8	1	0.00
PGN_0583	fig|431947.7.peg.577	1	0.00
PGN_1049	fig|431947.7.peg.1040	1	0.00
PGN_0894	fig|431947.7.peg.885	1	0.00

PGN_1797	fig|431947.7.peg.1771	1	0.00
PGN_0033	fig|431947.7.peg.30	1	0.00
PGN_1922	fig|431947.7.peg.1893	1	0.00
PGN_0728	fig|431947.7.peg.716	1	0.00
PGN_1643	fig|431947.7.peg.1620	1	0.00
	fig|431947.7.peg.862	1	0.03
PGN_0934	fig|431947.7.peg.923	1	0.00
PGN_1043	fig|431947.7.peg.1034	1	0.00
PGN_1726	fig|431947.7.peg.1701	1	0.01
PGN_1795	fig|431947.7.peg.1768	1	0.00
PGN_0350	fig|431947.7.peg.348	1	0.00
	fig|431947.7.peg.1372	1	0.00
PGN_0864	fig|431947.7.peg.854	1	0.00
PGN_1074	fig|431947.7.peg.1064	1	0.00
PGN_0179	fig|431947.7.peg.173	1	0.01
PGN_0184	fig|431947.7.peg.179	1	0.01
PGN_0608	fig|431947.7.peg.604	1	0.00
PGN_0694	fig|431947.7.peg.684	1	0.00
PGN_1073	fig|431947.7.peg.1063	1	0.04
PGN_1220	fig|431947.7.peg.1214	1	0.00
PGN_0400	fig|431947.7.peg.396	1	0.00
PGN_1069	fig|431947.7.peg.1059	1	0.02
PGN_0295	fig|431947.7.peg.292	1	0.00
PGN_1053	fig|431947.7.peg.1043	1	0.00
PGN_1518	fig|431947.7.peg.1501	1	0.00
PGN_0953	fig|431947.7.peg.943	1	0.00
PGN_0686	fig|431947.7.peg.676	1	0.00
PGN_0704	fig|431947.7.peg.694	1	0.00
PGN_1130	fig|431947.7.peg.1120	1	0.00
PGN_1314	fig|431947.7.peg.1302	1	0.01
PGN_1520	fig|431947.7.peg.1503	1	0.00
PGN_0567	fig|431947.7.peg.559	1	0.00
PGN_1387	fig|431947.7.peg.1375	1	0.00
PGN_1527	fig|431947.7.peg.1512	1	0.00
PGN_1557	fig|431947.7.peg.1540	1	0.00
PGN_0418	fig|431947.7.peg.417	1	0.00
PGN_0157	fig|431947.7.peg.151	1	0.01
PGN_0570	fig|431947.7.peg.562	1	0.00
	fig|431947.7.peg.555	1	0.03

PGN_0582	fig|431947.7.peg.576	1	0.02
PGN_1407	fig|431947.7.peg.1395	1	0.00
PGN_0386	fig|431947.7.peg.384	1	0.00
PGN_0428	fig|431947.7.peg.427	1	0.00
PGN_1748	fig|431947.7.peg.1724	1	0.00
PGN_1700	fig|431947.7.peg.1674	1	0.00
PGN_2081	fig|431947.7.peg.2045	1	0.00
PGN_1539	fig|431947.7.peg.1523	1	0.00
PGN_1536	fig|431947.7.peg.1520	1	0.00
PGN_1548	fig|431947.7.peg.1531	1	0.00
PGN_1067	fig|431947.7.peg.1057	1	0.01
	fig|431947.7.peg.1350	1	0.00
PGN_0123	fig|431947.7.peg.117	1	0.00
PGN_2082	fig|431947.7.peg.2046	1	0.00
PGN_0163	fig|431947.7.peg.158	1	0.02
PGN_0459	fig|431947.7.peg.459	1	0.00
PGN_1252	fig|431947.7.peg.1242	1	0.00
PGN_1050	fig|431947.7.peg.1041	1	0.00
PGN_0802	fig|431947.7.peg.791	1	0.00
PGN_0006	fig|431947.7.peg.6	1	0.00
PGN_1090	fig|431947.7.peg.1078	1	0.00
PGN_0211	fig|431947.7.peg.207	1	0.00
PGN_0300	fig|431947.7.peg.298	1	0.00
PGN_1796	fig|431947.7.peg.1769	1	0.00
PGN_1882	fig|431947.7.peg.1852	1	0.00
PGN_1481	fig|431947.7.peg.1463	1	0.00
PGN_0535	fig|431947.7.peg.531	1	0.00
PGN_0654	fig|431947.7.peg.645	1	0.00
PGN_0484	fig|431947.7.peg.483	1	0.00
PGN_0245	fig|431947.7.peg.243	1	0.00
PGN_1564	fig|431947.7.peg.1544	1	0.00
PGN_1528	fig|431947.7.peg.1513	1	0.00
PGN_1268	fig|431947.7.peg.1259	1	0.00
PGN_0683	fig|431947.7.peg.673	1	0.00
PGN_0614	fig|431947.7.peg.610	1	0.00
PGN_1516	fig|431947.7.peg.1499	1	0.01
PGN_1746	fig|431947.7.peg.1722	1	0.00
PGN_1402	fig|431947.7.peg.1391	1	0.00
PGN_0391	fig|431947.7.peg.389	1	0.00

PGN_0376	fig|431947.7.peg.374	1	0.00
PGN_1763	fig|431947.7.peg.1740	1	0.00
PGN_1745	fig|431947.7.peg.1721	1	0.00
PGN_1376	fig|431947.7.peg.1364	1	0.00
PGN_0247	fig|431947.7.peg.245	1	0.01
PGN_1979	fig|431947.7.peg.1943	1	0.00
PGN_1315	fig|431947.7.peg.1303	1	0.00
PGN_1697	fig|431947.7.peg.1671	1	0.00
PGN_2064	fig|431947.7.peg.2028	1	0.00
PGN_2001	fig|431947.7.peg.1967	1	0.00
PGN_0426	fig|431947.7.peg.425	1	0.00
PGN_0650	fig|431947.7.peg.643	1	0.00
PGN_0031	fig|431947.7.peg.28	1	0.00
PGN_1009	fig|431947.7.peg.999	1	0.01
PGN_0258	fig|431947.7.peg.257	1	0.00
	fig|431947.7.peg.906	1	0.00
PGN_1800	fig|431947.7.peg.1774	1	0.00
PGN_1401	fig|431947.7.peg.1390	1	0.00
PGN_0241	fig|431947.7.peg.239	1	0.00
PGN_1690	fig|431947.7.peg.1665	1	0.00
PGN_1616	fig|431947.7.peg.1594	1	0.01
PGN_1729	fig|431947.7.peg.1705	1	0.01
	fig|431947.7.peg.1389	1	0.00
PGN_1485	fig|431947.7.peg.1467	1	0.01
PGN_1992	fig|431947.7.peg.1958	1	0.01
PGN_0972	fig|431947.7.peg.963	1	0.01
PGN_0719	fig|431947.7.peg.707	1	0.04
PGN_1159	fig|431947.7.peg.1151	1	0.00
PGN_0248	fig|431947.7.peg.246	1	0.00
PGN_1819	fig|431947.7.peg.1792	1	0.04
PGN_1602	fig|431947.7.peg.1583	1	0.00
PGN_0968	fig|431947.7.peg.959	1	0.00
PGN_1977	fig|431947.7.peg.1941	1	0.00
PGN_0357	fig|431947.7.peg.357	1	0.00
PGN_0317	fig|431947.7.peg.314	1	0.00
PGN_1124	fig|431947.7.peg.1112	1	0.00
PGN_1899	fig|431947.7.peg.1870	1	0.04
PGN_0992	fig|431947.7.peg.981	1	0.02
	fig|431947.7.peg.736	1	0.01

PGN_1671	fig|431947.7.peg.1646	1	0.00
PGN_1016	fig|431947.7.peg.1006	1	0.02
PGN_0374	fig|431947.7.peg.372	1	0.00
PGN_1270	fig|431947.7.peg.1261	1	0.00
PGN_0314	fig|431947.7.peg.311	1	0.01
PGN_0685	fig|431947.7.peg.675	1	0.01
PGN_0358	fig|431947.7.peg.358	1	0.00
PGN_1058	fig|431947.7.peg.1048	1	0.03
PGN_0995	fig|431947.7.peg.984	1	0.01
PGN_1018	fig|431947.7.peg.1008	1	0.02
PGN_0889	fig|431947.7.peg.880	1	0.02
PGN_1601	fig|431947.7.peg.1582	1	0.00
PGN_1498	fig|431947.7.peg.1481	1	0.00
PGN_0917	fig|431947.7.peg.907	1	0.00
PGN_1113	fig|431947.7.peg.1099	1	0.00
PGN_1741	fig|431947.7.peg.1718	1	0.00
PGN_1038	fig|431947.7.peg.1029	1	0.00
PGN_1773	fig|431947.7.peg.1747	1	0.00
PGN_2026	fig|431947.7.peg.1990	1	0.01
	fig|431947.7.peg.1541	1	0.01
PGN_1358	fig|431947.7.peg.1347	1	0.02
PGN_1828	fig|431947.7.peg.1799	1	0.05
PGN_0221	fig|431947.7.peg.218	1	0.00
PGN_0531	fig|431947.7.peg.527	1	0.00
PGN_1040	fig|431947.7.peg.1031	1	0.04
PGN_1831	fig|431947.7.peg.1802	1	0.01
PGN_1834	fig|431947.7.peg.1805	1	0.00
PGN_0809	fig|431947.7.peg.798	1	0.00
PGN_0235	fig|431947.7.peg.232	1	0.00
PGN_1513	fig|431947.7.peg.1496	1	0.00
PGN_1269	fig|431947.7.peg.1260	1	0.01
PGN_1768	fig|431947.7.peg.1743	1	0.01
PGN_0465	fig|431947.7.peg.465	1	0.00
PGN_0259	fig|431947.7.peg.258	1	0.00
PGN_1699	fig|431947.7.peg.1673	1	0.00
PGN_2079	fig|431947.7.peg.2043	1	0.00
PGN_1437	fig|431947.7.peg.1422	1	0.00
PGN_1535	fig|431947.7.peg.1519	1	0.05
PGN_0733	fig|431947.7.peg.719	1	0.00

PGN_1873	fig|431947.7.peg.1843	1	0.01
PGN_0491	fig|431947.7.peg.489	1	0.01
PGN_1509	fig|431947.7.peg.1492	1	0.00
PGN_1966	fig|431947.7.peg.1931	1	0.00
PGN_1501	fig|431947.7.peg.1485	1	0.01
PGN_0935	fig|431947.7.peg.924	1	0.00
PGN_1561	fig|431947.7.peg.1542	1	0.02
PGN_1169	fig|431947.7.peg.1161	1	0.01
PGN_1304	fig|431947.7.peg.1294	1	0.00
PGN_1725	fig|431947.7.peg.1700	1	0.02
PGN_1500	fig|431947.7.peg.1484	1	0.03
PGN_1261	fig|431947.7.peg.1251	1	0.02
PGN_1565	fig|431947.7.peg.1545	1	0.01
PGN_1150	fig|431947.7.peg.1142	1	0.00
PGN_0736	fig|431947.7.peg.722	1	0.00
PGN_1625	fig|431947.7.peg.1602	1	0.00
PGN_0385	fig|431947.7.peg.383	1	0.03
PGN_0148	fig|431947.7.peg.141	1	0.01
PGN_0345	fig|431947.7.peg.342	1	0.00
PGN_1308	fig|431947.7.peg.1296	1	0.02
PGN_1617	fig|431947.7.peg.1595	1	0.01
PGN_1375	fig|431947.7.peg.1363	1	0.00
PGN_1730	fig|431947.7.peg.1706	1	0.03
PGN_1094	fig|431947.7.peg.1082	1	0.04
PGN_1227	fig|431947.7.peg.1221	1	0.02
PGN_0310	fig|431947.7.peg.307	1	0.01
PGN_1194	fig|431947.7.peg.1187	1	0.02
PGN_0537	fig|431947.7.peg.533	1	0.01
PGN_0638	fig|431947.7.peg.632	1	0.00
PGN_0282	fig|431947.7.peg.280	1	0.03
PGN_1991	fig|431947.7.peg.1957	1	0.03
PGN_1771	fig|431947.7.peg.1745	1	0.04
PGN_1878	fig|431947.7.peg.1849	1	0.05
PGN_1246	fig|431947.7.peg.1238	1	0.02
PGN_0015	fig|431947.7.peg.15	1	0.05
PGN_1969	fig|431947.7.peg.1935	1	0.05
PGN_1014	fig|431947.7.peg.1004	1	0.00
PGN_0705	fig|431947.7.peg.695	1	0.03
PGN_1600	fig|431947.7.peg.1581	1	0.00

PGN_0268	fig|431947.7.peg.267	1	0.03
PGN_0740	fig|431947.7.peg.726	1	0.02
PGN_0521	fig|431947.7.peg.517	1	0.00
PGN_1107	fig|431947.7.peg.1094	1	0.00
PGN_1998	fig|431947.7.peg.1964	0	0.00
PGN_1305	fig|431947.7.peg.1295	0	0.00
PGN_1833	fig|431947.7.peg.1804	0	0.01
PGN_0532	fig|431947.7.peg.528	0	0.00
PGN_0140	fig|431947.7.peg.134	0	0.00
	fig|431947.7.peg.1482	0	0.03
PGN_0647	fig|431947.7.peg.641	0	0.01
PGN_0642	fig|431947.7.peg.636	0	0.00
PGN_1158	fig|431947.7.peg.1150	0	0.01
PGN_1570	fig|431947.7.peg.1551	0	0.03
PGN_1362	fig|431947.7.peg.1351	0	0.04
PGN_1148	fig|431947.7.peg.1140	0	0.00
PGN_1103	fig|431947.7.peg.1090	0	0.03
	fig|431947.7.peg.1598	0	0.04
PGN_1002	fig|431947.7.peg.991	0	0.02
PGN_2003	fig|431947.7.peg.1968	0	0.01
PGN_0648	fig|431947.7.peg.642	0	0.05
PGN_1737	fig|431947.7.peg.1714	0	0.04
PGN_0462	fig|431947.7.peg.462	0	0.01
PGN_0201	fig|431947.7.peg.196	0	0.05
PGN_0125	fig|431947.7.peg.118	0	0.05
PGN_1549	fig|431947.7.peg.1532	0	0.04
PGN_1606	fig|431947.7.peg.1587	0	0.02
PGN_1128	fig|431947.7.peg.1118	0	0.03
PGN_1685	fig|431947.7.peg.1661	0	0.01
PGN_1537	fig|431947.7.peg.1521	0	0.05
PGN_0263	fig|431947.7.peg.262	0	0.04
PGN_1805	fig|431947.7.peg.1779	0	0.02
PGN_1143	fig|431947.7.peg.1135	0	0.04
PGN_1567	fig|431947.7.peg.1547	0	0.03
PGN_2024	fig|431947.7.peg.1988	0	0.03
PGN_1112	fig|431947.7.peg.1098	0	0.04
PGN_0335	fig|431947.7.peg.332	0	0.02
PGN_0865	fig|431947.7.peg.855	0	0.04
PGN_0590	fig|431947.7.peg.586	0	0.04

PGN_0987	fig|431947.7.peg.976	0	0.04
PGN_0962	fig|431947.7.peg.953	0	0.03
PGN_0487	fig|431947.7.peg.486	0	0.03
PGN_1782	fig|431947.7.peg.1755	0	0.04
PGN_1784	fig|431947.7.peg.1757	0	0.03
PGN_1891	fig|431947.7.peg.1862	0	0.04
PGN_0831	fig|431947.7.peg.819	0	0.03
PGN_1443	fig|431947.7.peg.1426	0	0.05
PGN_2021	fig|431947.7.peg.1985	0	0.03
PGN_0209	fig|431947.7.peg.204	0	0.01
PGN_0355	fig|431947.7.peg.355	0	0.05
PGN_0573	fig|431947.7.peg.565	0	0.03
PGN_0875	fig|431947.7.peg.866	0	0.03
PGN_1026	fig|431947.7.peg.1016	0	0.04
PGN_0646	fig|431947.7.peg.640	0	0.03
PGN_0788	fig|431947.7.peg.778	0	0.01
PGN_1970	fig|431947.7.peg.1936	0	0.02
PGN_0906	fig|431947.7.peg.896	0	0.05
PGN_0281	fig|431947.7.peg.279	0	0.01
PGN_1751	fig|431947.7.peg.1727	0	0.01
PGN_0546	fig|431947.7.peg.542	0	0.03
PGN_1702	fig|431947.7.peg.1676	0	0.04
PGN_0778	fig|431947.7.peg.768	0	0.03
PGN_0609	fig|431947.7.peg.605	0	0.05
PGN_1914	fig|431947.7.peg.1885	0	0.02
PGN_0743	fig|431947.7.peg.730	0	0.03
PGN_1655	fig|431947.7.peg.1632	0	0.03
PGN_2019	fig|431947.7.peg.1983	0	0.01
PGN_1964	fig|431947.7.peg.1929	0	0.02
PGN_1323	fig|431947.7.peg.1310	0	0.05
PGN_0410	fig|431947.7.peg.409	0	0.02
PGN_1079	fig|431947.7.peg.1070	0	0.03
PGN_1000	fig|431947.7.peg.989	0	0.01
	fig|431947.7.peg.408	0	0.01
PGN_1373	fig|431947.7.peg.1361	0	0.03
PGN_0068	fig|431947.7.peg.61	0	0.00
PGN_0829	fig|431947.7.peg.817	0	0.03
PGN_1141	fig|431947.7.peg.1132	0	0.02
PGN_1155	fig|431947.7.peg.1147	0	0.01

PGN_0353	fig|431947.7.peg.353	0	0.02
PGN_0114	fig|431947.7.peg.107	0	0.00
PGN_0658	fig|431947.7.peg.648	0	0.00
PGN_1571	fig|431947.7.peg.1552	-1	0.01
PGN_1654	fig|431947.7.peg.1631	-1	0.02
PGN_1976	fig|431947.7.peg.1940	-1	0.02
PGN_0116	fig|431947.7.peg.110	-1	0.00
PGN_1153	fig|431947.7.peg.1145	-1	0.01
PGN_1243	fig|431947.7.peg.1234	-1	0.04
PGN_0884	fig|431947.7.peg.875	-1	0.01
PGN_0742	fig|431947.7.peg.729	-1	0.03
PGN_1391	fig|431947.7.peg.1379	-1	0.01
PGN_0485	fig|431947.7.peg.484	-1	0.04
PGN_0311	fig|431947.7.peg.308	-1	0.02
PGN_0354	fig|431947.7.peg.354	-1	0.01
PGN_0759	fig|431947.7.peg.749	-1	0.05
PGN_0290	fig|431947.7.peg.288	-1	0.01
PGN_1162	fig|431947.7.peg.1154	-1	0.00
PGN_0675	fig|431947.7.peg.666	-1	0.03
PGN_1963	fig|431947.7.peg.1928	-1	0.01
PGN_0192	fig|431947.7.peg.187	-1	0.00
PGN_1753	fig|431947.7.peg.1729	-1	0.01
PGN_0665	fig|431947.7.peg.656	-1	0.03
PGN_0510	fig|431947.7.peg.506	-1	0.01
PGN_1430	fig|431947.7.peg.1415	-1	0.00
	fig|431947.7.peg.1945	-1	0.05
PGN_0299	fig|431947.7.peg.297	-1	0.00
PGN_1658	fig|431947.7.peg.1635	-1	0.00
PGN_1554	fig|431947.7.peg.1537	-1	0.03
PGN_0671	fig|431947.7.peg.662	-1	0.00
PGN_1444	fig|431947.7.peg.1427	-1	0.03
PGN_0667	fig|431947.7.peg.658	-1	0.01
PGN_1941	fig|431947.7.peg.1910	-1	0.00
PGN_2056	fig|431947.7.peg.2021	-1	0.01
PGN_0905	fig|431947.7.peg.895	-1	0.01
PGN_1968	fig|431947.7.peg.1934	-1	0.00
PGN_1139	fig|431947.7.peg.1130	-1	0.01
PGN_1010	fig|431947.7.peg.1000	-1	0.03
PGN_0867	fig|431947.7.peg.857	-1	0.04

PGN_1447	fig|431947.7.peg.1430	-1	0.00
PGN_0504	fig|431947.7.peg.501	-1	0.01
PGN_0059	fig|431947.7.peg.52	-1	0.03
PGN_0666	fig|431947.7.peg.657	-1	0.02
PGN_0811	fig|431947.7.peg.800	-1	0.01
PGN_1238	fig|431947.7.peg.1230	-1	0.02
PGN_0303	fig|431947.7.peg.301	-1	0.00
PGN_1320	fig|431947.7.peg.1308	-1	0.02
PGN_1942	fig|431947.7.peg.1911	-1	0.04
PGN_0001	fig|431947.7.peg.1	-1	0.01
PGN_1457	fig|431947.7.peg.1439	-1	0.00
PGN_1781	fig|431947.7.peg.1754	-1	0.01
PGN_0488	fig|431947.7.peg.487	-1	0.01
PGN_1236	fig|431947.7.peg.1229	-1	0.02
PGN_0096	fig|431947.7.peg.89	-1	0.00
PGN_1426	fig|431947.7.peg.1411	-1	0.04
PGN_0170	fig|431947.7.peg.166	-1	0.00
PGN_1318	fig|431947.7.peg.1306	-1	0.01
PGN_0012	fig|431947.7.peg.12	-1	0.02
PGN_1750	fig|431947.7.peg.1726	-1	0.00
PGN_1724	fig|431947.7.peg.1699	-1	0.03
PGN_0094	fig|431947.7.peg.87	-1	0.02
PGN_1962	fig|431947.7.peg.1927	-1	0.00
PGN_0814	fig|431947.7.peg.803	-1	0.00
PGN_0500	fig|431947.7.peg.496	-1	0.00
PGN_0340	fig|431947.7.peg.337	-1	0.02
PGN_0233	fig|431947.7.peg.230	-1	0.02
PGN_1755	fig|431947.7.peg.1731	-1	0.03
PGN_1876	fig|431947.7.peg.1847	-1	0.02
PGN_1152	fig|431947.7.peg.1144	-1	0.02
PGN_0141	fig|431947.7.peg.135	-1	0.00
PGN_0154	fig|431947.7.peg.149	-1	0.00
PGN_0503	fig|431947.7.peg.500	-1	0.00
PGN_1283	fig|431947.7.peg.1275	-1	0.02
PGN_1022	fig|431947.7.peg.1012	-1	0.02
PGN_0309	fig|431947.7.peg.306	-1	0.02
PGN_0283	fig|431947.7.peg.281	-1	0.01
PGN_0360	fig|431947.7.peg.360	-1	0.00
PGN_1466	fig|431947.7.peg.1447	-1	0.00

PGN_0813	fig|431947.7.peg.802	-1	0.00
PGN_0396	fig|431947.7.peg.393	-1	0.00
PGN_1415	fig|431947.7.peg.1401	-1	0.01
PGN_1254	fig|431947.7.peg.1244	-1	0.02
PGN_1357	fig|431947.7.peg.1345	-1	0.00
PGN_0792	fig|431947.7.peg.782	-1	0.00
PGN_1659	fig|431947.7.peg.1636	-1	0.00
PGN_0251	fig|431947.7.peg.249	-1	0.00
PGN_0997	fig|431947.7.peg.986	-1	0.04
PGN_0378	fig|431947.7.peg.376	-1	0.00
PGN_0805	fig|431947.7.peg.794	-1	0.00
PGN_0830	fig|431947.7.peg.818	-1	0.01
PGN_1446	fig|431947.7.peg.1429	-1	0.00
PGN_1648	fig|431947.7.peg.1626	-1	0.00
PGN_0501	fig|431947.7.peg.497	-1	0.00
PGN_0519	fig|431947.7.peg.515	-1	0.04
PGN_2010	fig|431947.7.peg.1975	-1	0.00
PGN_1033	fig|431947.7.peg.1023	-1	0.00
PGN_1347	fig|431947.7.peg.1334	-1	0.00
PGN_0765	fig|431947.7.peg.755	-1	0.01
PGN_1475	fig|431947.7.peg.1456	-1	0.01
PGN_1647	fig|431947.7.peg.1625	-1	0.00
PGN_1597	fig|431947.7.peg.1578	-1	0.02
PGN_0804	fig|431947.7.peg.793	-1	0.01
PGN_0044	fig|431947.7.peg.39	-1	0.00
PGN_0815	fig|431947.7.peg.804	-1	0.00
PGN_1488	fig|431947.7.peg.1470	-1	0.01
PGN_0076	fig|431947.7.peg.69	-1	0.02
PGN_0243	fig|431947.7.peg.241	-1	0.00
PGN_1205	fig|431947.7.peg.1198	-1	0.00
PGN_1324	fig|431947.7.peg.1311	-1	0.04
PGN_0035	fig|431947.7.peg.32	-1	0.00
PGN_1343	fig|431947.7.peg.1330	-1	0.00
PGN_0414	fig|431947.7.peg.413	-1	0.02
PGN_0416	fig|431947.7.peg.415	-1	0.01
PGN_0528	fig|431947.7.peg.524	-1	0.01
PGN_0509	fig|431947.7.peg.505	-1	0.00
PGN_1122	fig|431947.7.peg.1110	-1	0.00
PGN_0912	fig|431947.7.peg.901	-1	0.00

PGN_0828	fig|431947.7.peg.816	-1	0.00
PGN_0791	fig|431947.7.peg.781	-1	0.00
PGN_0342	fig|431947.7.peg.339	-1	0.04
PGN_0255	fig|431947.7.peg.254	-1	0.00
PGN_0207	fig|431947.7.peg.202	-1	0.00
PGN_0543	fig|431947.7.peg.539	-1	0.00
PGN_1012	fig|431947.7.peg.1002	-1	0.01
PGN_0709	fig|431947.7.peg.699	-1	0.00
PGN_0133	fig|431947.7.peg.127	-1	0.03
PGN_0134	fig|431947.7.peg.128	-1	0.03
PGN_0002	fig|431947.7.peg.2	-1	0.00
PGN_1961	fig|431947.7.peg.1926	-1	0.00
PGN_0883	fig|431947.7.peg.874	-1	0.00
PGN_1062	fig|431947.7.peg.1052	-1	0.00
	fig|431947.7.peg.2014	-1	0.00
	fig|431947.7.peg.213	-1	0.00
PGN_1271	fig|431947.7.peg.1262	-1	0.01
PGN_0533	fig|431947.7.peg.529	-1	0.00
PGN_1209	fig|431947.7.peg.1203	-1	0.02
PGN_1377	fig|431947.7.peg.1365	-1	0.00
PGN_0167	fig|431947.7.peg.163	-1	0.00
PGN_1892	fig|431947.7.peg.1863	-1	0.01
PGN_1140	fig|431947.7.peg.1131	-1	0.00
PGN_2055	fig|431947.7.peg.2020	-1	0.00
	fig|431947.7.peg.1733	-1	0.03
PGN_0869	fig|431947.7.peg.859	-1	0.00
PGN_1469	fig|431947.7.peg.1449	-1	0.00
PGN_1511	fig|431947.7.peg.1494	-1	0.01
PGN_1776	fig|431947.7.peg.1750	-1	0.01
PGN_1174	fig|431947.7.peg.1166	-1	0.00
PGN_0977	fig|431947.7.peg.968	-1	0.00
PGN_1282	fig|431947.7.peg.1274	-1	0.01
PGN_1317	fig|431947.7.peg.1305	-1	0.00
	fig|431947.7.peg.440	-1	0.00
PGN_1470	fig|431947.7.peg.1450	-1	0.01
PGN_1902	fig|431947.7.peg.1873	-1	0.02
PGN_1138	fig|431947.7.peg.1129	-1	0.01
PGN_1201	fig|431947.7.peg.1194	-1	0.02
PGN_0965	fig|431947.7.peg.956	-1	0.00

PGN_2066	fig|431947.7.peg.2030	-1	0.01
PGN_1489	fig|431947.7.peg.1471	-1	0.00
PGN_0887	fig|431947.7.peg.879	-1	0.00
PGN_1676	fig|431947.7.peg.1652	-1	0.00
PGN_0544	fig|431947.7.peg.540	-1	0.01
PGN_2008	fig|431947.7.peg.1973	-1	0.00
PGN_0710	fig|431947.7.peg.700	-1	0.00
PGN_0379	fig|431947.7.peg.377	-1	0.00
PGN_1886	fig|431947.7.peg.1856	-1	0.00
PGN_1754	fig|431947.7.peg.1730	-1	0.00
PGN_0118	fig|431947.7.peg.112	-1	0.00
PGN_0807	fig|431947.7.peg.796	-1	0.01
PGN_0635	fig|431947.7.peg.629	-1	0.00
PGN_0806	fig|431947.7.peg.795	-1	0.00
PGN_1461	fig|431947.7.peg.1443	-1	0.03
PGN_0470	fig|431947.7.peg.470	-1	0.00
PGN_0274	fig|431947.7.peg.273	-1	0.00
PGN_1756	fig|431947.7.peg.1732	-1	0.00
PGN_0078	fig|431947.7.peg.70	-1	0.00
	fig|431947.7.peg.1859	-1	0.05
PGN_0893	fig|431947.7.peg.884	-1	0.00
PGN_1947	fig|431947.7.peg.1916	-1	0.00
PGN_1967	fig|431947.7.peg.1933	-1	0.00
PGN_0693	fig|431947.7.peg.683	-1	0.00
PGN_1179	fig|431947.7.peg.1171	-1	0.00
PGN_1512	fig|431947.7.peg.1495	-1	0.00
	fig|431947.7.peg.1437	-1	0.02
	fig|431947.7.peg.894	-1	0.02
PGN_0634	fig|431947.7.peg.628	-1	0.00
PGN_1326	fig|431947.7.peg.1313	-1	0.02
PGN_1474	fig|431947.7.peg.1455	-1	0.01
PGN_0691	fig|431947.7.peg.681	-1	0.01
PGN_0193	fig|431947.7.peg.188	-1	0.02
PGN_0986	fig|431947.7.peg.974	-1	0.04
PGN_0696	fig|431947.7.peg.686	-1	0.01
PGN_0066	fig|431947.7.peg.59	-1	0.04
PGN_1349	fig|431947.7.peg.1336	-1	0.00
PGN_0868	fig|431947.7.peg.858	-1	0.04
PGN_0191	fig|431947.7.peg.186	-1	0.00

PGN_0902	fig|431947.7.peg.891	-1	0.00
PGN_1348	fig|431947.7.peg.1335	-1	0.00
PGN_1117	fig|431947.7.peg.1104	-1	0.00
PGN_1572	fig|431947.7.peg.1553	-1	0.00
PGN_1384	fig|431947.7.peg.1371	-1	0.00
PGN_0672	fig|431947.7.peg.663	-1	0.00
PGN_1178	fig|431947.7.peg.1170	-1	0.00
PGN_1325	fig|431947.7.peg.1312	-1	0.00
PGN_0080	fig|431947.7.peg.72	-1	0.00
PGN_0315	fig|431947.7.peg.312	-1	0.00
	fig|431947.7.peg.1386	-1	0.00
PGN_0891	fig|431947.7.peg.882	-1	0.00
PGN_1723	fig|431947.7.peg.1698	-1	0.00
PGN_0904	fig|431947.7.peg.893	-1	0.00
PGN_1850	fig|431947.7.peg.1820	-1	0.00
PGN_1694	fig|431947.7.peg.1669	-1	0.00
PGN_0996	fig|431947.7.peg.985	-1	0.00
PGN_0534	fig|431947.7.peg.530	-1	0.00
PGN_0677	fig|431947.7.peg.668	-1	0.01
PGN_0206	fig|431947.7.peg.201	-1	0.00
PGN_1629	fig|431947.7.peg.1606	-1	0.04
PGN_1974	fig|431947.7.peg.1938	-1	0.00
PGN_1029	fig|431947.7.peg.1019	-1	0.05
PGN_0250	fig|431947.7.peg.248	-1	0.00
PGN_0377	fig|431947.7.peg.375	-1	0.00
PGN_0711	fig|431947.7.peg.701	-1	0.00
PGN_0812	fig|431947.7.peg.801	-1	0.00
PGN_0625	fig|431947.7.peg.619	-1	0.00
PGN_0781	fig|431947.7.peg.771	-1	0.00
PGN_0746	fig|431947.7.peg.733	-1	0.01
PGN_1095	fig|431947.7.peg.1083	-1	0.00
PGN_1025	fig|431947.7.peg.1015	-1	0.00
PGN_0547	fig|431947.7.peg.543	-1	0.00
PGN_2052	fig|431947.7.peg.2017	-1	0.00
PGN_0674	fig|431947.7.peg.665	-1	0.00
PGN_0827	fig|431947.7.peg.815	-1	0.00
PGN_1035	fig|431947.7.peg.1025	-1	0.00
PGN_0119	fig|431947.7.peg.113	-1	0.00
PGN_0231	fig|431947.7.peg.228	-1	0.01

PGN_1172	fig|431947.7.peg.1164	-1	0.00
PGN_0556	fig|431947.7.peg.550	-1	0.00
PGN_1195	fig|431947.7.peg.1188	-1	0.00
	fig|431947.7.peg.883	-1	0.01
PGN_0880	fig|431947.7.peg.871	-1	0.00
PGN_0083	fig|431947.7.peg.76	-1	0.00
PGN_1167	fig|431947.7.peg.1159	-1	0.00
PGN_1618	fig|431947.7.peg.1596	-1	0.00
PGN_1088	fig|431947.7.peg.1076	-1	0.00
	fig|431947.7.peg.403	-1	0.03
PGN_0852	fig|431947.7.peg.844	-1	0.00
PGN_1230	fig|431947.7.peg.1224	-1	0.00
PGN_0169	fig|431947.7.peg.165	-1	0.00
PGN_2086	fig|431947.7.peg.2051	-1	0.00
PGN_0307	fig|431947.7.peg.304	-1	0.00
	fig|431947.7.peg.88	-1	0.03
PGN_0779	fig|431947.7.peg.769	-1	0.00
PGN_1674	fig|431947.7.peg.1649	-1	0.00
PGN_0515	fig|431947.7.peg.511	-1	0.00
PGN_1848	fig|431947.7.peg.1818	-1	0.00
PGN_0826	fig|431947.7.peg.814	-1	0.00
	fig|431947.7.peg.1121	-1	0.02
PGN_1808	fig|431947.7.peg.1782	-1	0.00
PGN_0626	fig|431947.7.peg.620	-1	0.01
PGN_0514	fig|431947.7.peg.510	-1	0.00
PGN_1170	fig|431947.7.peg.1162	-1	0.00
PGN_0415	fig|431947.7.peg.414	-1	0.00
PGN_0911	fig|431947.7.peg.900	-1	0.00
PGN_0689	fig|431947.7.peg.679	-1	0.04
PGN_0782	fig|431947.7.peg.772	-1	0.01
PGN_0964	fig|431947.7.peg.955	-1	0.00
PGN_0117	fig|431947.7.peg.111	-1	0.00
PGN_2017	fig|431947.7.peg.1981	-1	0.00
PGN_0898	fig|431947.7.peg.888	-1	0.00
PGN_0468	fig|431947.7.peg.468	-1	0.00
PGN_0038	fig|431947.7.peg.34	-1	0.00
PGN_0780	fig|431947.7.peg.770	-1	0.00
PGN_2007	fig|431947.7.peg.1972	-1	0.00
	fig|431947.7.peg.1783	-1	0.04

PGN_0527	fig|431947.7.peg.523	-1	0.00
PGN_1086	fig|431947.7.peg.1074	-1	0.03
	fig|431947.7.peg.975	-1	0.02
PGN_0673	fig|431947.7.peg.664	-1	0.00
	fig|431947.7.peg.108	-1	0.00
PGN_1302	fig|431947.7.peg.1292	-1	0.00
PGN_2061	fig|431947.7.peg.2026	-1	0.00
PGN_0726	fig|431947.7.peg.714	-1	0.00
PGN_0411	fig|431947.7.peg.410	-1	0.00
PGN_1468	fig|431947.7.peg.1448	-1	0.00
PGN_0881	fig|431947.7.peg.872	-1	0.00
PGN_1011	fig|431947.7.peg.1001	-1	0.00
PGN_0226	fig|431947.7.peg.224	-1	0.00
PGN_0989	fig|431947.7.peg.978	-1	0.00
PGN_1743	fig|431947.7.peg.1719	-1	0.00
PGN_1530	fig|431947.7.peg.1515	-1	0.00
PGN_0356	fig|431947.7.peg.356	-1	0.01
PGN_0998	fig|431947.7.peg.987	-1	0.00
PGN_1637	fig|431947.7.peg.1614	-1	0.01
PGN_0988	fig|431947.7.peg.977	-1	0.00
	fig|431947.7.peg.2003	-1	0.04
PGN_1367	fig|431947.7.peg.1357	-1	0.00
PGN_0277	fig|431947.7.peg.276	-1	0.02
PGN_0627	fig|431947.7.peg.621	-1	0.00
PGN_0469	fig|431947.7.peg.469	-1	0.00
PGN_1458	fig|431947.7.peg.1440	-1	0.00
PGN_1204	fig|431947.7.peg.1197	-1	0.00
PGN_0882	fig|431947.7.peg.873	-1	0.00
	fig|431947.7.peg.1479	-1	0.00
PGN_0981	fig|431947.7.peg.970	-1	0.00
PGN_0190	fig|431947.7.peg.185	-1	0.00
PGN_1973	fig|431947.7.peg.1937	-1	0.00
PGN_1529	fig|431947.7.peg.1514	-1	0.00
PGN_1166	fig|431947.7.peg.1158	-1	0.00
	fig|431947.7.peg.1651	-1	0.00
PGN_2078	fig|431947.7.peg.2042	-1	0.00
PGN_0082	fig|431947.7.peg.75	-1	0.00
PGN_1632	fig|431947.7.peg.1609	-1	0.00
PGN_0223	fig|431947.7.peg.221	-1	0.00

PGN_1173	fig|431947.7.peg.1165	-1	0.00
PGN_1034	fig|431947.7.peg.1024	-1	0.00
PGN_1788	fig|431947.7.peg.1761	-1	0.00
PGN_1698	fig|431947.7.peg.1672	-1	0.00
PGN_0471	fig|431947.7.peg.471	-1	0.00
PGN_1344	fig|431947.7.peg.1331	-1	0.00
PGN_0412	fig|431947.7.peg.411	-1	0.00
PGN_1677	fig|431947.7.peg.1653	-1	0.00
PGN_0963	fig|431947.7.peg.954	-1	0.00
PGN_1462	fig|431947.7.peg.1444	-1	0.00
PGN_1764	fig|431947.7.peg.1741	-1	0.00
PGN_0990	fig|431947.7.peg.979	-1	0.00
PGN_0692	fig|431947.7.peg.682	-1	0.00
PGN_0276	fig|431947.7.peg.275	-1	0.02
PGN_1587	fig|431947.7.peg.1568	-1	0.00
PGN_2054	fig|431947.7.peg.2019	-1	0.00
PGN_1163	fig|431947.7.peg.1155	-1	0.00
PGN_1165	fig|431947.7.peg.1157	-1	0.00
PGN_1576	fig|431947.7.peg.1557	-1	0.00
PGN_1675	fig|431947.7.peg.1650	-1	0.00
PGN_1840	fig|431947.7.peg.1810	-1	0.00
PGN_0758	fig|431947.7.peg.748	-1	0.00
PGN_1080	fig|431947.7.peg.1071	-1	0.00
PGN_1176	fig|431947.7.peg.1168	-1	0.00
PGN_0152	fig|431947.7.peg.146	-1	0.00
PGN_0724	fig|431947.7.peg.712	-1	0.00
PGN_1496	fig|431947.7.peg.1478	-1	0.00
PGN_0766	fig|431947.7.peg.756	-1	0.00
PGN_1313	fig|431947.7.peg.1301	-1	0.00
	fig|431947.7.peg.1245	-1	0.00
PGN_0628	fig|431947.7.peg.622	-1	0.00
PGN_0622	fig|431947.7.peg.616	-1	0.00
PGN_1345	fig|431947.7.peg.1332	-1	0.00
PGN_0725	fig|431947.7.peg.713	-1	0.00
PGN_1573	fig|431947.7.peg.1554	-1	0.00
PGN_1631	fig|431947.7.peg.1608	-1	0.01
PGN_1256	fig|431947.7.peg.1247	-1	0.00
PGN_0940	fig|431947.7.peg.929	-1	0.00
PGN_1021	fig|431947.7.peg.1011	-1	0.00

PGN_0777	fig|431947.7.peg.767	-1	0.00
PGN_0975	fig|431947.7.peg.966	-1	0.00
PGN_1842	fig|431947.7.peg.1812	-1	0.00
PGN_1849	fig|431947.7.peg.1819	-1	0.00
PGN_2080	fig|431947.7.peg.2044	-1	0.00
PGN_0870	fig|431947.7.peg.860	-1	0.00
PGN_1151	fig|431947.7.peg.1143	-1	0.00
PGN_0572	fig|431947.7.peg.564	-1	0.00
PGN_2053	fig|431947.7.peg.2018	-1	0.00
PGN_1949	fig|431947.7.peg.1918	-1	0.00
PGN_1223	fig|431947.7.peg.1217	-1	0.00
PGN_1390	fig|431947.7.peg.1378	-1	0.00
PGN_1789	fig|431947.7.peg.1762	-1	0.00
PGN_0115	fig|431947.7.peg.109	-1	0.00
PGN_0228	fig|431947.7.peg.226	-1	0.00
PGN_1240	fig|431947.7.peg.1232	-1	0.00
PGN_1790	fig|431947.7.peg.1763	-1	0.00
PGN_0279	fig|431947.7.peg.278	-1	0.00
PGN_0571	fig|431947.7.peg.563	-1	0.00
PGN_0067	fig|431947.7.peg.60	-1	0.00
PGN_1346	fig|431947.7.peg.1333	-1	0.00
PGN_1734	fig|431947.7.peg.1710	-1	0.00
PGN_1846	fig|431947.7.peg.1816	-1	0.00
PGN_1845	fig|431947.7.peg.1815	-1	0.00
PGN_0976	fig|431947.7.peg.967	-1	0.00
PGN_1255	fig|431947.7.peg.1246	-1	0.00
PGN_1574	fig|431947.7.peg.1555	-1	0.00
PGN_1844	fig|431947.7.peg.1814	-1	0.00
PGN_1577	fig|431947.7.peg.1558	-1	0.00
PGN_1333	fig|431947.7.peg.1320	-1	0.04
PGN_1691	fig|431947.7.peg.1666	-1	0.00
PGN_1777	fig|431947.7.peg.1751	-1	0.00
PGN_0225	fig|431947.7.peg.223	-1	0.00
PGN_1575	fig|431947.7.peg.1556	-1	0.00
PGN_0481	fig|431947.7.peg.480	-1	0.00
PGN_0974	fig|431947.7.peg.965	-1	0.00
PGN_0607	fig|431947.7.peg.603	-1	0.00
PGN_0023	fig|431947.7.peg.21	-1	0.00
PGN_1851	fig|431947.7.peg.1821	-1	0.00

PGN_1673	fig|431947.7.peg.1648	-1	0.00
PGN_1120	fig|431947.7.peg.1108	-1	0.00
PGN_1588	fig|431947.7.peg.1569	-1	0.00
PGN_1787	fig|431947.7.peg.1760	-1	0.00
PGN_0099	fig|431947.7.peg.92	-1	0.00
PGN_1847	fig|431947.7.peg.1817	-1	0.00
PGN_1841	fig|431947.7.peg.1811	-1	0.00
PGN_1853	fig|431947.7.peg.1823	-1	0.00
PGN_0549	fig|431947.7.peg.545	-1	0.00
PGN_0545	fig|431947.7.peg.541	-1	0.00
PGN_1505	fig|431947.7.peg.1489	-1	0.00
PGN_2009	fig|431947.7.peg.1974	-1	0.00
PGN_1945	fig|431947.7.peg.1914	-1	0.00
PGN_1852	fig|431947.7.peg.1822	-1	0.00
PGN_1843	fig|431947.7.peg.1813	-1	0.00
PGN_0508	fig|431947.7.peg.504	-1	0.00
PGN_1054	fig|431947.7.peg.1044	-2	0.00
PGN_1759	fig|431947.7.peg.1736	-2	0.00
	fig|431947.7.peg.91	-2	0.00
PGN_2051	fig|431947.7.peg.2016	-2	0.00
PGN_0482	fig|431947.7.peg.481	-2	0.00
PGN_0612	fig|431947.7.peg.608	-2	0.00
PGN_1389	fig|431947.7.peg.1377	-2	0.00
PGN_0610	fig|431947.7.peg.606	-2	0.00
PGN_0224	fig|431947.7.peg.222	-2	0.00
PGN_0361	fig|431947.7.peg.361	-2	0.00
PGN_1590	fig|431947.7.peg.1571	-2	0.00
PGN_0024	fig|431947.7.peg.22	-2	0.00
PGN_1242	fig|431947.7.peg.1233	-2	0.00
PGN_0278	fig|431947.7.peg.277	-2	0.00
PGN_0308	fig|431947.7.peg.305	-2	0.00
PGN_1855	fig|431947.7.peg.1825	-2	0.00
PGN_1872	fig|431947.7.peg.1842	-2	0.00
PGN_1633	fig|431947.7.peg.1610	-2	0.00
PGN_0624	fig|431947.7.peg.618	-2	0.00
PGN_1506	fig|431947.7.peg.1490	-2	0.00
PGN_1854	fig|431947.7.peg.1824	-2	0.00
PGN_0548	fig|431947.7.peg.544	-2	0.00
PGN_1239	fig|431947.7.peg.1231	-2	0.00

PGN_0553	fig|431947.7.peg.547	-2	0.04
PGN_1905	fig|431947.7.peg.1876	-2	0.00
PGN_0611	fig|431947.7.peg.607	-2	0.00
PGN_1856	fig|431947.7.peg.1826	-2	0.00
PGN_1871	fig|431947.7.peg.1841	-2	0.00
PGN_0723	fig|431947.7.peg.711	-2	0.00
	fig|431947.7.peg.73	-2	0.00
PGN_1121	fig|431947.7.peg.1109	-2	0.00
PGN_1857	fig|431947.7.peg.1827	-2	0.00
PGN_1868	fig|431947.7.peg.1838	-2	0.00
PGN_0623	fig|431947.7.peg.617	-2	0.00
PGN_1870	fig|431947.7.peg.1840	-2	0.00
PGN_1222	fig|431947.7.peg.1216	-2	0.00
PGN_1692	fig|431947.7.peg.1667	-2	0.00
PGN_1869	fig|431947.7.peg.1839	-2	0.00
PGN_1867	fig|431947.7.peg.1837	-2	0.00
PGN_1761	fig|431947.7.peg.1738	-2	0.00
PGN_1862	fig|431947.7.peg.1832	-2	0.00
PGN_1859	fig|431947.7.peg.1829	-2	0.00
PGN_1589	fig|431947.7.peg.1570	-2	0.00
PGN_1738	fig|431947.7.peg.1715	-2	0.00
PGN_1760	fig|431947.7.peg.1737	-2	0.00
PGN_1369	fig|431947.7.peg.1358	-2	0.00
PGN_1858	fig|431947.7.peg.1828	-2	0.00
PGN_1866	fig|431947.7.peg.1836	-2	0.00
PGN_0074	fig|431947.7.peg.67	-2	0.02
PGN_0668	fig|431947.7.peg.659	-2	0.00
PGN_1861	fig|431947.7.peg.1831	-2	0.00
PGN_1865	fig|431947.7.peg.1835	-2	0.00
PGN_0901	fig|431947.7.peg.890	-2	0.00
PGN_0558	fig|431947.7.peg.552	-2	0.01
PGN_1762	fig|431947.7.peg.1739	-2	0.00
PGN_1863	fig|431947.7.peg.1833	-2	0.00
PGN_1864	fig|431947.7.peg.1834	-2	0.00
PGN_1860	fig|431947.7.peg.1830	-2	0.00
PGN_1215	fig|431947.7.peg.1209	-2	0.02
